# Effect of Respiratory Syncytial Virus on the Global Burden of Lower Respiratory Infections: Lessons From the Global Burden of Disease Study 1990–2021

**DOI:** 10.1002/iid3.70415

**Published:** 2026-04-23

**Authors:** Jiafen Zhao, Tao Zhou, Cheng Jiang, Feng Peng

**Affiliations:** ^1^ Department of Rehabilitation Xiantao First People's Hospital Xiantao China; ^2^ Department of Gastroenterology and Hepatology, Huanggang Hospital of Traditional Chinese Medicine Hubei University of Chinese Medicine Huanggang China; ^3^ Department of Respiratory Medicine Hubei Provincial Hospital of Traditional Chinese Medicine/Affiliated Hospital of Hubei University of Chinese Medicine Wuhan Hubei China; ^4^ Hubei Shizhen Laboratory Wuhan China; ^5^ Department of Pediatric, Xiangyang Hospital of Traditional Chinese Medicine (Xiangyang Institute of Traditional Chinese Medicine) Hubei University of Chinese Medicine Xiangyang China

**Keywords:** DALYs, disease burden, GBD 2021, lower respiratory infections, respiratory syncytial virus

## Abstract

**Background:**

Respiratory syncytial virus (RSV) is a major cause of lower respiratory infections (LRIs), especially in children, the elderly, and immunocompromised individuals.

**Methods:**

Using GBD 1990–2021 data, we analyzed global RSV‐related LRI deaths and DALYs. Trends were assessed via age‐standardized rates and joinpoint regression. We also examined regional and demographic disparities and their association with the Sociodemographic Index (SDI).

**Results:**

In 2021, RSV‐related LRIs caused 31,525.1 deaths and 2.59 million DALYs globally, marking a 78.9% decrease since 1990. The greatest decline occurred between 2020 and 2021. South Asia and Sub‐Saharan Africa showed the highest burden. Children under 5 and the elderly (80+) were most affected, particularly males. A negative correlation was observed between SDI and disease burden.

**Conclusions:**

RSV burden has declined, but remains high in low‐SDI regions. Sustained public health efforts and vaccination are critical to further reduce impact.

## Summary

- RSV‐related LRIs burden globally declined by 78.9% from 1990 to 2021, with a significant drop between 2020 and 2021, likely due to COVID‐19 interventions.
- South Asia and Sub‐Saharan Africa have the highest age‐standardized death and DALY rates, reflecting ongoing regional disparities.
- Higher Sociodemographic Index (SDI) countries generally faced lower burdens, though low‐SDI regions with weak healthcare systems remain vulnerable.
- Infants, young children, and the elderly are most affected by RSV, with males consistently experiencing higher disease burden.
- The potential for post‐pandemic resurgence highlights the need for continued public health efforts, including vaccination and healthcare access improvements.

## 1 Introduction

Respiratory syncytial virus (RSV) is a negative‐sense RNA virus, with a non‐segmented genome encoding 11 proteins, and belongs to the Paramyxoviridae family. Structurally, RSV is characterized by a lipid bilayer envelope encasing a nucleocapsid [1]. This virus poses a significant therapeutic challenge, primarily due to its narrow treatment window—antiviral therapy is most effective only when administered early in the course of infection [2]. Furthermore, the absence of both effective antiviral drugs and safe, efficacious vaccines, particularly for high‐risk populations, complicates disease management [3, 4]. As a leading etiological agent of lower respiratory infections (LRIs), RSV has a disproportionate impact on vulnerable groups, such as infants, the elderly, and individuals with compromised immune systems [5, 6]. In 2019, RSV was responsible for approximately 3.6% of all global deaths in children aged 28 days to 6 months, with 97% of these fatalities occurring in low‐ and middle‐income countries, which bear the majority of RSV‐related mortality [6]. Despite the substantial burden posed by RSV, assessing its full global impact remains challenging due to regional disparities in prevalence, healthcare infrastructure, and underlying risk factors [7, 8].

Several studies [6, 9, 10] have provided critical epidemiological data on RSV‐related LRIs, though they have predominantly focused on the burden of LRIs in children under the age of 5 years, with limited exploration of the disease's impact across other age groups. The most recent analysis of RSV‐attributable LRIs, utilizing data from the Global Burden of Disease (GBD) 2019, systematically examined the current burden and temporal trends of RSV‐associated LRIs based on age, sex, geographic location, and socioeconomic status [11, 12]. However, numerous studies have indicated that the epidemiology and disease burden of RSV have been substantially reshaped by the COVID‐19 pandemic, with large‐scale non‐pharmaceutical interventions (NPIs) affecting the transmission dynamics of RSV and other respiratory infections [13, 14]. Moreover, the rates of RSV‐associated LRIs exhibited varied trajectories in different income regions in 2021 [13, 15]. To date, the global epidemiological characteristics of RSV‐induced LRI in 2021 remain underexplored. To address this knowledge gap, we analyzed the number of deaths and age‐standardized death rates (ASDRs) associated with RSV‐induced LRIs in 204 countries and territories from 1990 to 2021 using data from the GBD 2021.

The GBD 2021, a globally integrated database with advanced analytical tools, provides the most recent and comprehensive framework for assessing disease burden, encompassing data from 204 countries and territories over a 30‐year period. This initiative represents a culmination of long‐term international collaboration among governments [16]. In this study, we present an updated analysis of the epidemiological trends of RSV‐associated LRIs at global, regional, and national levels based on the latest data from the GBD 2021. Our findings are intended to inform global health policy, guide resource allocation, and support the development of effective public health strategies aimed at addressing the ongoing burden of RSV‐related LRIs.

## 2 Method Details

### 2.1 Data Sources

The latest Global Burden of Disease 2021 study provides comprehensive estimates of epidemiological data on the burden of 371 diseases and injuries from 1990 to 2021 in 204 countries and territories and 21 regions. Detailed descriptions of the methodologies have been reported, and fatal and non‐fatal estimates have been published (https://vizhub.healthdata.org/gbd-compare/ and https://ghdx.healthdata.org/gbd-results-tool). In the GBD, estimates of incidence, DALYs, and mortality are given for each disease (namely RSV‐related LRIs).

### 2.2 DALYs (Disability‐Adjusted Life Years)

DALYs are a critical metric used to quantify the total loss of healthy life years due to diseases, combining both years of life lost (YLLs) and years lived with disability (YLDs). YLLs represent the years lost due to premature death, while YLDs measure the impact of living with a disability or health condition. In contrast to standard life expectancy, which measures average life span, DALYs reflect both fatal and non‐fatal health outcomes, offering a more comprehensive view of the burden of disease. The disability weight, ranging from 0 (representing perfect health) to 1 (representing death), plays a central role in calculating YLDs. By multiplying the number of people living with a particular condition by the corresponding disability weight, DALYs are calculated to represent the overall health loss from both premature death and ongoing disability. This metric is widely used in global health assessments to determine the overall impact of diseases, injuries, and health conditions on populations.

### 2.3 SDI (Socio‐Demographic Index)

The Socio‐Demographic Index (SDI) is a composite measure introduced by the Institute for Health Metrics and Evaluation (IHME) to assess the socio‐economic development of countries and regions. It reflects the interconnectedness between social development factors and health outcomes. SDI is calculated as the geometric mean of three key indicators: the average educational attainment of people aged 15 and older, the total fertility rate among women under 25 years, and the lagged distribution of per capita income. These components provide a holistic view of a region's socio‐economic status, with a value ranging from 0 to 1. The world is divided into five SDI quintiles: low SDI (0 < 0.46), low‐middle SDI (0.46 ≤ 0.61), middle SDI (0.61 ≤ 0.69), high‐middle SDI (0.69 ≤ 0.81), and high SDI (0.81 ≤ 1.00). This stratification helps to analyze health and disease burden across different socio‐economic contexts, allowing for a more nuanced understanding of how socio‐economic conditions influence health outcomes across 204 countries and territories [17].

### 2.4 Statistical Analysis

The burden of RSV‐related LRIs was quantified using age‐standardized rates (ASRs) to account for variations in population age structures. ASRs were calculated using the World Health Organization's standard population, with the formula: $A\mathrm{SR}=\frac{\sum_{i=1}^{A}a_{i}w_{i}}{\sum_{i=1}^{A}w_{i}}\times$100,000. where $a_{i}$ is the age‐specific rate for the ⅈ‐th age group, and $w_{i}$ is the number of individuals in that age group, with the sum across all age groups normalized to the standard population. To assess temporal trends, we used generalized linear regression models to calculate the estimated annual percentage change (EAPC) in ASRs from 1990 to 2021. The EAPC and its 95% confidence interval (CI) were derived from the regression coefficient (*β*): *EAPC* = 100 × [exp(*β*)−1]. The burden of RSV‐related LRIs was further analyzed in terms of incidence, mortality, and DALYs for the years 1990 and 2021, across various regions. The results were presented as ASRs to allow comparison across different regions, sex, and age groups.

We also assessed inequalities in the burden of RSV‐related LRIs using the Slope Index of Inequality and the Concentration Index. These indices reflect absolute and relative gradients of inequality, respectively, and were linked to the SDI. Regression models in R were used for the calculation of these indices.

Finally, temporal trends in RSV‐related LRIs from 1990 to 2021 were analyzed using joinpoint regression models. These models help identify significant changes in the trend of disease burden over time, with the annual percentage change (APC) calculated for each segment of the data. The formula for APC is: *APC* = (exp(*β*)−1) × 100. In addition, the Average Annual Percentage Change (AAPC) was calculated, providing a summary of the overall trend across the entire period. This was done by weighting the APCs from each segment by the width of the intervals, allowing for an integrated view of the disease trends.

Statistical analyses were performed using Joinpoint 5.1.0.0 (April 2024), R software 4.3.2.

## 3 Results

### 3.1 Global Level

**Figure 1:**
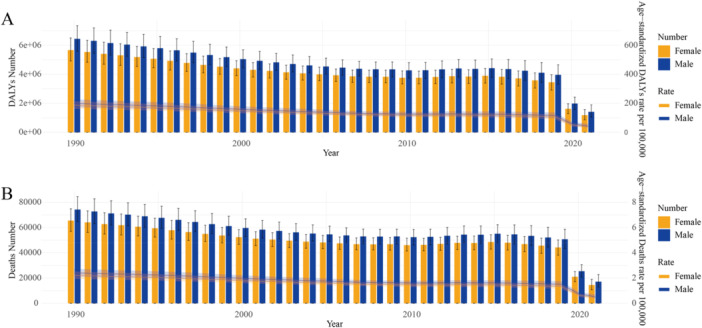
The trends in numbers and ASRs of DALYs (A) and deaths (B) for RSV‐related LRIs from 1990 to 2021. Error bars indicate the 95% uncertainty interval for numbers. Shading indicates the 95% uncertainty interval for rates.

In 2021, 31,525.1 deaths from RSV‐related LRIs were reported globally, with an age‐standardized rate of 487.5, a decrease of 78.9% since 1990. The number of DALYs for RSV‐related LRIs globally was 2.59 million, with an age‐standardized rate of 40,821.6 DALYs, a 78.9% decrease since 1990 (Table 1). The number of deaths and age‐standardized death rates have generally declined from 1990 to 2021, with a particularly noticeable drop in 2020 and 2021. Males consistently exhibit higher death numbers and rates than females (Figure 1B). Similarly, the number of DALYs and age‐standardized DALY rates also show a downward trend over time, with the most significant decline occurring in 2020 and 2021. Once again, males experience a higher number and rate of DALYs compared to females (Figure 1A). Overall, there is a clear downward trend in both deaths and DALYs, with the most substantial reductions observed in 2020 and 2021, and males continue to bear a higher disease burden than females.

**Table 1:** Deaths, and disability adjusted life years (DALYs) for RSV‐related LRIs in 2021, and percentage change in age‐standardised rates (ASRs) per 100, by Global Burden of Disease region, from 1990 to 2021.

Characteristics	Deaths (95% UI)			DALYs (95% UI)		
	Number of Deaths (95% UI)	ASRs per 100 (95% UI)	Percentage_change in ASRs from 1990 to 2021	Number of Deaths (95% UI)	ASRs per 100 (95% UI)	Percentage_change in ASRs from 1990 to 2021
Global	31525.1 (23348.3 to 41871.4)	487.5 (360 to 648.8)	−78.9 (−84.1 to −72.9)	2591507.3 (1902003.7 to 3468792.1)	40821.6 (29911.7 to 54593.6)	−78.9 (−84.2 to −72.9)
High‐income Asia Pacific	2.4 (0.2 to 12.5)	0.4 (0 to 2.1)	−99.9 (−100 to −99.6)	32.5 (3.2 to 168)	11.8 (1.3 to 55.5)	−99.9 (−100 to −99.7)
High‐income North America	113.8 (55.9 to 205.8)	21.8 (10.4 to 39.4)	−92.8 (−96.5 to −87)	3149.2 (1488.4 to 5682.6)	957.3 (447.6 to 1737)	−91.9 (−96.1 to −85.5)
Western Europe	97.8 (62.1 to 143.4)	9.5 (6 to 13.9)	−96.5 (−97.8 to −94.9)	1547.8 (974.9 to 2258)	283.1 (180.6 to 413.8)	−97.2 (−98.2 to −95.9)
Australasia	0.5 (0.1 to 1.2)	1.1 (0.3 to 2.4)	−99.5 (−99.9 to −99)	11.2 (2.9 to 25)	39.6 (9.9 to 90.4)	−99.6 (−99.9 to −99.2)
Andean Latin America	284.7 (107 to 464.9)	484.4 (181.9 to 789)	−82.5 (−93.4 to −70)	16774.3 (6358.1 to 28740.1)	27778.3 (10527.1 to 47550.7)	−87.7 (−95.3 to −77.6)
Tropical Latin America	263.7 (78.1 to 586.7)	124.8 (36.5 to 277.7)	−90.9 (−97.3 to −79.3)	12370.7 (3599.8 to 27803.9)	6581.3 (1926.2 to 14881)	−94 (−98.2 to −85.9)
Central Latin America	212.4 (142.4 to 305.1)	104.7 (69.9 to 150.9)	−91.9 (−94.6 to −88)	15563.7 (10261.8 to 22529.6)	7931.5 (5232.5 to 11475.6)	−92.4 (−94.9 to −88.7)
Southern Latin America	32 (14 to 62)	42.4 (18.4 to 82.6)	−92.8 (−96.8 to −86)	919.6 (398.5 to 1779.1)	1668.9 (736.4 to 3259.4)	−95.6 (−98.1 to −91.6)
Caribbean	2.4 (0.7 to 11.8)	5.6 (1.7 to 28)	−99.6 (−99.9 to −98.2)	161.4 (46.6 to 808.9)	412.4 (118.6 to 2062.4)	−99.7 (−99.9 to −98.4)
Central Europe	9.5 (2.8 to 22.8)	6.7 (2.2 to 14.9)	−99.3 (−99.8 to −98.4)	302.7 (103 to 665.8)	363 (132.4 to 755.5)	−99.5 (−99.8 to −99)
Eastern Europe	68.4 (29.6 to 130.6)	38 (16.3 to 72.2)	−94.4 (−97.6 to −89.2)	3395.6 (1479.6 to 6438)	2545.7 (1093.2 to 4889.7)	−95.6 (−98.1 to −91.6)
Central Asia	222.5 (118.5 to 365.4)	229.1 (123 to 375.4)	−95.1 (−97.3 to −91.9)	19064.4 (9879 to 31308.3)	19290 (10024.8 to 31667.4)	−95.3 (−97.5 to −92.3)
North Africa and Middle East	1039.1 (560.2 to 1796.8)	198.3 (110.6 to 337.6)	−90.8 (−95.1 to −83.7)	79031.2 (40379.2 to 138867.8)	13736.7 (7076.3 to 24077.9)	−92.6 (−96.3 to −86.3)
South Asia	11561.3 (5798.1 to 18693.9)	774.3 (387.4 to 1250.5)	−70.6 (−85.3 to −52.2)	959423.9 (484294.9 to 1553206.5)	63003.2 (31787.4 to 102034)	−72.4 (−86.1 to −55.4)
Southeast Asia	398.6 (259.4 to 584.9)	73 (47.6 to 107.5)	−96.5 (−97.8 to −94.8)	27229.2 (17728.7 to 40560.7)	4892.4 (3195.6 to 7283.7)	−97.2 (−98.3 to −95.7)
East Asia	908.3 (513.4 to 1516.2)	98.4 (55.4 to 163.6)	−95.8 (−97.7 to −92.8)	45581.4 (25631 to 76212.4)	6675.1 (3694.2 to 11116.3)	−96.6 (−98.2 to −94.3)
Oceania	23.9 (11.5 to 43.8)	123.7 (59.4 to 224.1)	−95.9 (−98.1 to −93)	2105.4 (1010.7 to 3885)	10512.4 (5047.6 to 19343)	−95.9 (−98.1 to −93)
Western Sub‐Saharan Africa	9996.3 (5877.2 to 14968.9)	1342.1 (797.9 to 2000.7)	−70 (−80.9 to −56.2)	872819.4 (513360.7 to 1305845.6)	108549.6 (63846.5 to 162598)	−71.4 (−81.7 to −58.4)
Eastern Sub‐Saharan Africa	4505.3 (2917 to 6559)	848.7 (570.9 to 1234.9)	−74.3 (−83.6 to −63.9)	384101.8 (246486.4 to 559266.9)	62476.8 (40511.1 to 90898.2)	−77.1 (−85.3 to −67.7)
Central Sub‐Saharan Africa	1537.3 (729.6 to 2437.4)	975.9 (484.2 to 1496.6)	−70.5 (−84.6 to −54.6)	128479.8 (59859.3 to 204950.2)	65724.5 (31333.4 to 103937.9)	−75.9 (−87.7 to −62.7)
Southern Sub‐Saharan Africa	245 (98.1 to 490.2)	332 (133.4 to 665.6)	−84.6 (−93.8 to −69.5)	19442.2 (7745.8 to 39009.6)	24962.5 (9962.8 to 50122.6)	−85.5 (−94.2 to −71.4)

*Note:* 95% UI=95% uncertainty intervals.

### 3.2 Regional Level

In 2021, South Asia (11,561.3), Western Sub‐Saharan Africa (9996.3), and Eastern Sub‐Saharan Africa (4505.3) had the highest age‐standardized point deaths for RSV‐related LRIs, whereas Australasia (0.5), High‐income Asia Pacific (2.4), and the Caribbean (2.4) had the lowest. South Asia (959,423.9), Western Sub‐Saharan Africa (872,819.4), and Eastern Sub‐Saharan Africa (384,101.8) had the highest age‐standardized DALY rates from RSV‐related LRIs in 2021, with the lowest rates in Australasia (11.2), High‐income Asia Pacific (32.5), and the Caribbean (161.4). The largest decreases in the age‐standardized point deaths of RSV‐related LRIs, from 1990 to 2021, were found in High‐income Asia Pacific (−99.9%), Caribbean (−99.6%), and Central Europe (−99.3%). In the same period, all regions showed a decrease in the age‐standardized DALY rates from RSV‐related LRIs, with the largest decreases in High‐income Asia Pacific (−99.9%), Caribbean (−99.7%), and Australasia (−99.6%) (Table 1).

### 3.3 National Level

**Figure 2:**
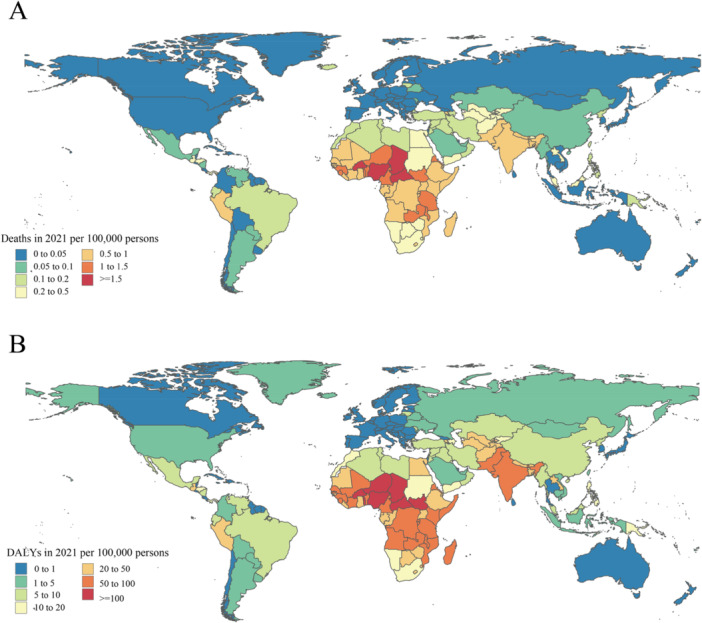
The global disease burden of RSV‐related LRIs per 100,000 population in 2021, by country. (A) Age‐standardised death rate in 2021. (B) Age standardised DALYs rate in 2021.

In 2021, the national age‐standardized DALY rate of RSV‐related LRIs ranged from 0.1 to 1450.3 patients per 1,000. The highest rates were seen in Burkina Faso (1450.3), Central African Republic (1,396.5), and the Republic of San Marino (1238.5), whereas the lowest rates were in the Republic of Korea (0.1), Japan (0.1), and Brunei Darussalam (0.2) (Figure 2 and Supporting Information S2: Table S1). The percentage change in the age‐standardized point DALY, from 1990 to 2021, all national levels showed a decrease in the age‐standardized DALY rates from RSV‐related LRIs, with the largest decreases in the Republic of Korea (−100%), Japan (−99.9%), North Macedonia (−99.9%), and Brunei Darussalam (−99.9%) (Supporting Information S2: Table S2).

### 3.4 Age and Sex Patterns

**Figure 3:**
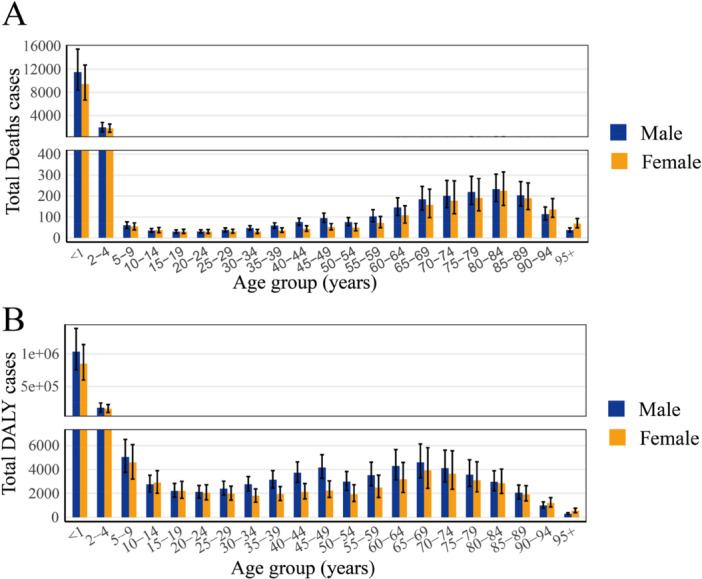
Number of total Deaths (A) and DALYs (B) cases globally of RSV‐related LRIs, by age and sex in 2021.

In 2021, the total DALY cases and deaths are shown across various age groups and genders. In both cases, the highest numbers are observed in the youngest age groups (< 1 and 2–4 years), with a notable decline in DALYs and deaths as age increases. Men generally experience higher DALY and death rates than women, particularly in the older age groups. While the total DALY cases decrease with age, they remain relatively high in men up to the age group of 70–74, after which they decline (Figure 3B). In contrast, death cases peak in the 80‐84 age group and remain higher in men across all age groups, particularly in the elderly population (> 85 years). These trends indicate a higher burden of RSV‐related LRIs in younger children and elderly individuals, with a more significant impact on men overall (Figure 3A).

### 3.5 Association With the Sociodemographic Index

**Figure 4:**
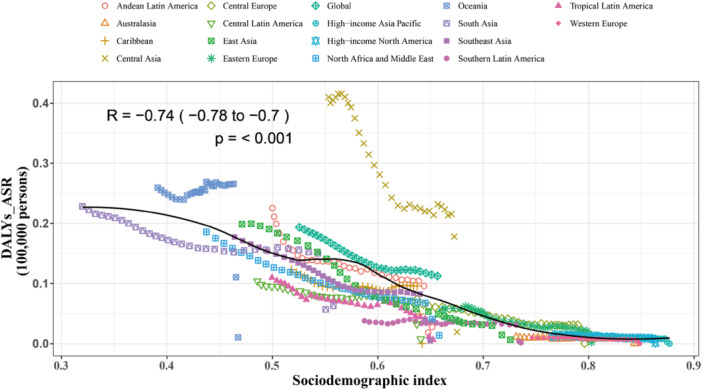
Age standardised DALYs rate of RSV‐related LRIs for the 21 Global Burden of Disease regions by sociodemographic index, 1990–2021. Thirty points are plotted for each region and show the observed age‐standardised DALYs rate from 1990 to 2021 for that region. Expected values, based on sociodemographic index and disease rates in all locations, are shown as a solid line. Regions above the solid line represent a higher than expected burden (e.g., Central Asia) and regions below the line show a lower than expected burden (e.g., Tropical Latin America).

At the regional level, the age‐standardized DALY rate for RSV‐related LRIs shows a negative correlation with the SDI, with a correlation coefficient of *R* = −0.74 and a *p*‐value of < 0.001, indicating a statistically significant relationship, from 1990 to 2021. The data reveals a clear downward trend, suggesting that as the SDI increases, the burden of RSV‐related LRIs (measured in DALY rates per 100,000 persons) decreases. Specifically, regions such as Eastern Europe, South Asia, Southeast Asia, and Southern sub‐Saharan Africa have higher‐than‐expected DALY rates despite having a higher SDI, while regions like High‐income Asia Pacific, Western Europe, North Africa and the Middle East, and Tropical Latin America show lower‐than‐expected DALY rates. This may be attributed to better healthcare systems and disease prevention measures in these regions. This trend indicates that more developed regions with higher SDI typically experience lower burdens of RSV‐related LRIs, although some areas still face higher burdens, possibly due to disparities in public health policies and resource distribution (Figure 4).

At the country level, in 2021, the burden of RSV‐related LRIs showed a negative correlation with socioeconomic development. This relationship is reflected by an *R* value of −0.75 (with a 95% confidence interval between −0.81 and −0.68) and a *p*‐value of 0.000, indicating a highly significant association. The data reveals a clear downward trend in the DALY rates as the SDI increases. Specifically, countries and territories such as Somalia, Niger, Chad, and South Sudan have much higher‐than‐expected burdens of RSV‐related LRIs, whereas countries like Norway, Denmark, Iceland, and Germany have much lower‐than‐expected burdens despite having higher SDI values. This trend suggests that while socioeconomic development generally leads to a decrease in RSV‐related LRIs, some regions still face disproportionately high burdens, possibly due to gaps in healthcare infrastructure and disease prevention efforts (Figure S1).

### 3.6 Jointpoint Analysis From 1990 to 2021

**Figure 5:**
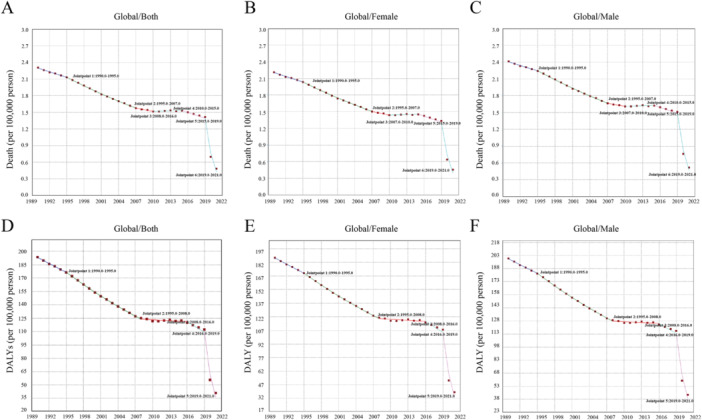
Joinpoint regression results of global trends for Deaths and DALYs of RSV‐related LRIs. (A–C) Present the global trends in mortality (both sexes, females, and males), while (D–F) present the global trends in DALYs (both sexes, females, and males), all based on Joinpoint regression analysis.

Joinpoint regression was utilized to estimate the temporal trends of ASDR for RSV‐related LRIs globally. Over the past 30 years, the global burden has gradually decreased, but the trend was not consistent. A slow downward trend was observed from 1990 to 1995, followed by a period of more rapid decline from 1995 to 2008, a brief stabilization from 2008 to 2016, and then a sharper decline from 2019 to 2021. Specifically, the Annual Percent Change (APC) during 1990–1995 was −1.79%, followed by −2.69% from 1995 to 2008, −0.29% from 2008 to 2016, and −3.27% from 2016 to 2019, with a sharp decline of −42.72% observed from 2019 to 2021. The global ASDR trends were similar for both males and females, with males experiencing a slightly larger reduction (−5.2683, 95% UI: −5.6835 to −4.8512) compared to females (−5.5182, 95% UI: −5.9303 to −5.1044) (Figure 5 and Supporting Information S2: Table S3).

## 4 Discussion

Our analysis of the global burden of RSV‐related LRIs from 1990 to 2021 highlights several important trends in the mortality and morbidity associated with this virus. Despite a significant reduction in the overall burden of RSV‐related LRIs, the disease remains a major public health concern, particularly in low‐ and middle‐income regions and among vulnerable populations. This study provides comprehensive insights into the temporal patterns, regional disparities, and demographic factors that influence the burden of RSV, offering valuable guidance for future public health interventions.

The global decline in deaths and DALYs due to RSV‐related LRIs from 1990 to 2021, particularly the sharp reduction observed in 2020‐2021, can be partially attributed to the indirect effects of the COVID‐19 pandemic. The widespread implementation of NPIs, such as physical distancing and enhanced hygiene measures, likely reduced the transmission of RSV and other respiratory infections [18, 19, 20, 21]. The marked drop in disease burden during this period underscores the vulnerability of RSV to public health measures designed to limit respiratory virus spread. However, it is crucial to recognize that while these measures have contributed to short‐term reductions in RSV transmission, the long‐term impact of COVID‐19 on RSV epidemiology remains uncertain and warrants further investigation.

Regionally, our findings reveal stark disparities in the burden of RSV‐related LRIs, with South Asia, Eastern Sub‐Saharan Africa, and Western Sub‐Saharan Africa bearing the highest age‐standardized death rates and DALY rates. These regions have historically struggled with inadequate healthcare infrastructure, limited access to vaccines and antiviral therapies, and high rates of malnutrition, all of which exacerbate the impact of RSV on vulnerable populations. In contrast, regions such as High‐income Asia Pacific, Australasia, and the Caribbean have witnessed more substantial declines in RSV‐related mortality and morbidity, likely due to better healthcare access, improved disease prevention strategies, and more robust public health systems. These findings highlight the importance of regional healthcare disparities and the need for tailored interventions that address the specific challenges faced by low‐ and middle‐income countries.

At the national level, we observed that countries with higher SDI scores generally experienced lower RSV‐related disease burdens, suggesting that socioeconomic development plays a critical role in mitigating the impact of RSV. However, there were notable exceptions, with countries such as Somalia and Chad exhibiting disproportionately high rates of RSV‐related LRIs despite their low SDI. These outliers emphasize the need for a nuanced understanding of the relationship between socio‐economic development and disease burden, as factors such as healthcare system capacity, access to preventive services, and regional epidemiological patterns may also influence disease outcomes.

Our analysis also underscores the age‐ and sex‐related patterns of RSV burden. The highest rates of DALYs and deaths were observed in infants and young children, as well as in the elderly, particularly those aged 80 years and older. These findings are consistent with previous studies highlighting the vulnerability of these age groups to RSV infections [22, 23, 24]. Notably, men consistently exhibited higher rates of both deaths and DALYs compared to women across all age groups, a pattern that warrants further exploration. The sex‐based differences in disease burden may reflect underlying biological, social, or environmental factors that influence susceptibility to RSV, and future research should aim to better understand the mechanisms driving these disparities.

The joinpoint regression analysis revealed a gradual decline in the global age‐standardized death rate for RSV‐related LRIs over the past three decades, with the most significant decrease occurring between 2019 and 2021. This decline aligns with the broader trends observed in other respiratory infections, which have also seen reductions in mortality due to improvements in healthcare, vaccination efforts, and disease prevention strategies. However, the COVID‐19 pandemic's impact on RSV epidemiology raises concerns about the long‐term sustainability of these improvements. As public health measures are relaxed and populations return to pre‐pandemic behaviors, there is a risk of a resurgence in RSV‐related infections, particularly in regions with insufficient healthcare infrastructure.

In conclusion, while the global burden of RSV‐related LRIs has decreased substantially over the past three decades, the disease remains a significant health threat, particularly in low‐income regions and among vulnerable populations. Our findings highlight the need for continued efforts to reduce RSV‐associated morbidity and mortality through targeted interventions, such as improving healthcare access, expanding vaccination programs, and enhancing disease prevention strategies. Given the observed regional disparities and the potential for a post‐pandemic resurgence, it is crucial for policymakers to prioritize RSV in their public health agendas, particularly in areas with high disease burdens and limited resources. Furthermore, continuous surveillance and in‐depth research are vital for tracking the evolving epidemiology of RSV and guiding the development of effective interventions to mitigate its global health impact. As the world transitions beyond the COVID‐19 pandemic, sustained monitoring and strategic policy prioritization will be essential to maintaining progress in reducing RSV‐related morbidity and mortality.

## Author Contributions

Jiafen Zhao designed the study, performed the data analysis, and drafted the manuscript. Tao Zhou contributed to data analysis and methodology and revised the manuscript. Cheng Jiang was involved in methodological development, interpretation of results, and manuscript writing. Feng Peng conceived the study and supervised the project, secured funding, and critically reviewed and revised the manuscript. All authors read and approved the final version of the manuscript.

## Funding

This work received support from Hubei Provincial Natural Science Foundation Joint Project (2025AFD115); Hubei Provincial Administration of Traditional Chinese Medicine General Project (ZY2025L271); Hubei Provincial Natural Science Foundation General Project (2024AFB908).

## Ethics Statement

The human participant studies were reviewed and approved using data from the Global Health Data Exchange, which did not mandate informed patient consent. The study employed an anonymized, publicly available dataset that did not include any personal identifying details of the participants. In line with national laws and institutional guidelines, written informed consent for participation was not required for this research.

## Conflicts of Interest

The authors declare no conflicts of interest.

## Supporting information

Supporting Figure

Supporting Table
