# Supplementary material for: Effect of Respiratory Syncytial Virus on the Global Burden of Lower Respiratory Infections: Lessons From the Global Burden of Disease Study 1990–2021
Source: Immun Inflamm Dis. 2026 Apr 23;14(4):e70415. doi: 10.1002/iid3.70415 (PMC13106949; doi:10.1002/iid3.70415)
Supplement: Supplementary file 1 — Supporting Figure [file IID3-14-e70415-s002.docx]

**Fig S1.**


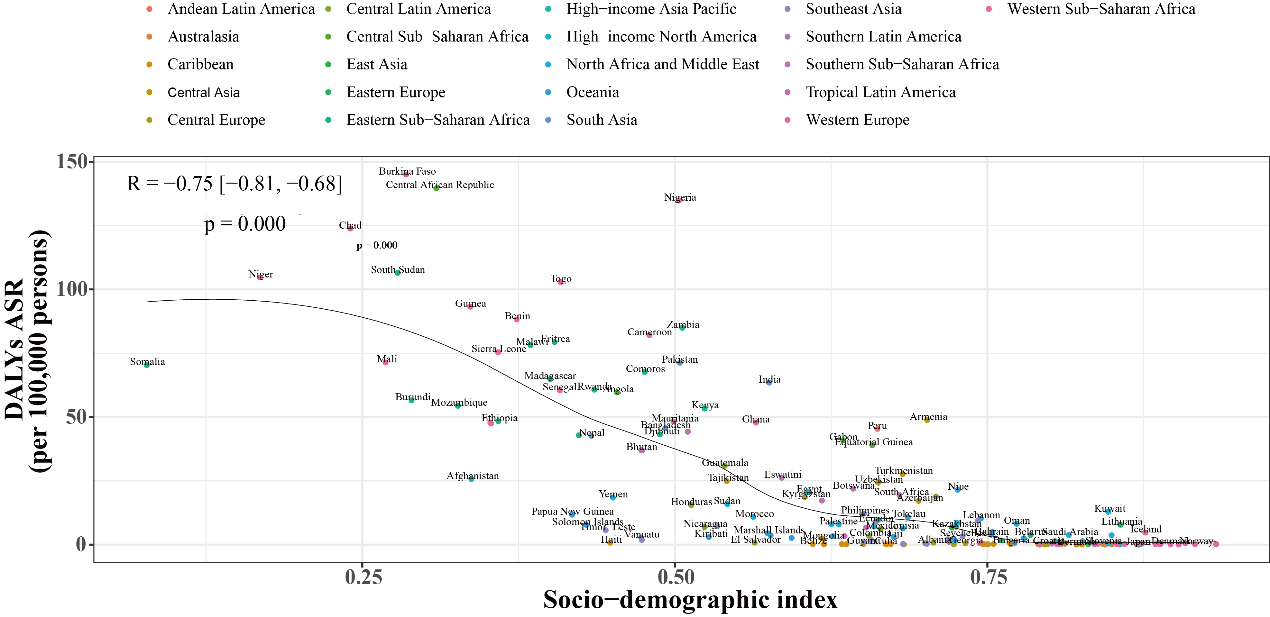


Fig S1. Age-standardised DALYs rate of RSV-related LRIs for 204 countries and territories, by SDI, in 2021; Expected values based on the Socio-demographic Index and disease rates in all locations are shown as the black line. Each point shows the observed age standardised DALYs rate for each country in 202
