# Supplementary material for: Effect of Respiratory Syncytial Virus on the Global Burden of Lower Respiratory Infections: Lessons From the Global Burden of Disease Study 1990–2021
Source: Immun Inflamm Dis. 2026 Apr 23;14(4):e70415. doi: 10.1002/iid3.70415 (PMC13106949; doi:10.1002/iid3.70415)
Supplement: Supplementary file 2 — Supporting Table [file IID3-14-e70415-s001.docx]

| Table S1. DALYs of RSV-related LRIs in 1990 and 2021 and the percentage change in the age-standardised rates (ASRs) per 1000, by location | | | | | |
| --- | --- | --- | --- | --- | --- |
|  | 1990 | | 2021 | | Percentage change in the |
|  | No (95% UI) | ASRs per 1000 (95% UI) | No (95% UI) | ASRs per 1000 (95% UI) | ASRs per 1000 |
| Australia | 1225 (1141,1312) | 89.9 (83.2,96.7) | 9 (2,19) | 0.3 (0.1,0.8) | -99.6 (-99.9,-99.1) |
| New Zealand | 617 (569,667) | 204.6 (188.4,222.2) | 3 (1,6) | 0.6 (0.2,1.5) | -99.7 (-99.9,-99.3) |
| Antigua and Barbuda | 14 (12,17) | 239.7 (202.7,282.2) | 0 (0,0) | 1.7 (0.5,8.1) | -99.3 (-99.8,-96.8) |
| Barbados | 55 (47,64) | 263 (220.3,303.8) | 0 (0,1) | 1.4 (0.4,7.2) | -99.5 (-99.8,-97.3) |
| Belize | 231 (205,262) | 793 (704.4,895.7) | 1 (0,4) | 2.1 (0.6,9.9) | -99.7 (-99.9,-98.8) |
| Bermuda | 6 (6,7) | 135.9 (117.6,157.4) | 0 (0,0) | 0.3 (0.1,1.7) | -99.7 (-99.9,-98.8) |
| Dominican Republic | 7692 (6221,9173) | 747.1 (606,887.9) | 15 (4,70) | 1.4 (0.4,6.8) | -99.8 (-100,-99.1) |
| Grenada | 57 (47,69) | 511 (423.9,615.5) | 0 (0,1) | 1.8 (0.6,8.6) | -99.6 (-99.9,-98.3) |
| Jamaica | 696 (613,795) | 257.6 (227.4,293.2) | 2 (1,8) | 0.9 (0.3,4.3) | -99.7 (-99.9,-98.4) |
| Puerto Rico | 773 (722,826) | 239.1 (223,255.5) | 1 (0,7) | 0.8 (0.2,3.6) | -99.7 (-99.9,-98.5) |
| Saint Kitts and Nevis | 22 (20,25) | 502.6 (450.6,563.1) | 0 (0,0) | 2.5 (0.8,12) | -99.5 (-99.8,-97.7) |
| Saint Lucia | 49 (40,58) | 301.7 (253,356) | 0 (0,1) | 1.4 (0.4,6.9) | -99.5 (-99.9,-97.7) |
| Saint Vincent and the Grenadines | 43 (36,54) | 364.7 (301,445.7) | 0 (0,1) | 1.4 (0.4,6.8) | -99.6 (-99.9,-98.2) |
| United States Virgin Islands | 20 (15,25) | 185 (141.2,231.7) | 0 (0,0) | 0.4 (0.1,2) | -99.8 (-99.9,-98.9) |
| Georgia | 12883 (11202,14752) | 3015.6 (2618,3457.3) | 13 (3,42) | 4.8 (1.1,15.6) | -99.8 (-100,-99.5) |
| Mongolia | 16059 (13211,18969) | 4709.2 (3878.7,5555.9) | 89 (6,385) | 24 (1.6,103.2) | -99.5 (-100,-97.8) |
| Turkmenistan | 29461 (26000,33950) | 4960.3 (4379.5,5713.9) | 1476 (661,2653) | 276.6 (123.8,497.3) | -94.4 (-97.4,-89.6) |
| Bosnia and Herzegovina | 555 (445,678) | 163.2 (130.1,200.2) | 2 (1,5) | 1.1 (0.4,2.5) | -99.3 (-99.8,-98.4) |
| Hungary | 1525 (1398,1668) | 231.8 (210.9,255.8) | 9 (4,20) | 1.4 (0.5,2.9) | -99.4 (-99.8,-98.7) |
| Montenegro | 138 (104,181) | 275.8 (207.7,364.4) | 0 (0,1) | 0.9 (0.3,1.9) | -99.7 (-99.9,-99.3) |
| North Macedonia | 1315 (1070,1641) | 798 (649.3,996.4) | 1 (1,3) | 1.2 (0.4,2.4) | -99.9 (-100,-99.7) |
| Romania | 31382 (29374,33223) | 1982.5 (1853.9,2099.7) | 55 (7,195) | 4.9 (0.7,17.5) | -99.8 (-100,-99.1) |
| Central African Republic | 21314 (14363,28775) | 3851.2 (2638.9,5164.4) | 11802 (5403,19793) | 1396.5 (645.7,2342.9) | -63.7 (-82.9,-38.8) |
| Democratic Republic of the Congo | 208430 (140829,279452) | 2647.3 (1815.9,3518.9) | 81648 (38060,133910) | 653.2 (310.7,1064.5) | -75.3 (-87.6,-60.1) |
| Democratic People's Republic of Korea | 31303 (23554,40876) | 1251.9 (943.6,1628.7) | 1086 (530,1920) | 68.4 (32.9,122.2) | -94.5 (-97.5,-89.4) |
| Taiwan (Province of China) | 5031 (4724,5329) | 319.2 (300.2,338.3) | 742 (412,1230) | 47.7 (26.2,79.4) | -85.1 (-91.8,-75.2) |
| Republic of Moldova | 5402 (4734,6191) | 1375.6 (1204.9,1577.1) | 121 (40,247) | 69.1 (22.7,141.6) | -95 (-98.4,-89.6) |
| Russian Federation | 63630 (60891,66541) | 620.5 (593.5,649.2) | 2462 (628,5538) | 25.4 (6.4,57.1) | -95.9 (-99,-90.8) |
| Ukraine | 15543 (13398,17807) | 454.3 (388.9,522.5) | 401 (197,677) | 16.5 (8.2,26.5) | -96.4 (-98.2,-93.9) |
| United Republic of Tanzania | 187324 (147129,230039) | 3572.5 (2808.6,4365.3) | 78032 (43713,127517) | 903 (507.7,1473.1) | -74.7 (-85.1,-59) |
| Brunei Darussalam | 49 (41,58) | 179.3 (156.4,208.1) | 0 (0,0) | 0.2 (0,1) | -99.9 (-100,-99.5) |
| Japan | 14595 (13687,15429) | 140.3 (133.4,146.5) | 28 (1,160) | 0.1 (0,0.7) | -99.9 (-100,-99.5) |
| Republic of Korea | 6101 (4942,7526) | 190.8 (153.8,234.9) | 1 (1,1) | 0 (0,0) | -100 (-100,-100) |
| Canada | 2688 (2511,2843) | 113.2 (106.3,119.9) | 135 (32,339) | 3.3 (0.8,8.5) | -97.1 (-99.3,-92.6) |
| Greenland | 20 (16,25) | 365.6 (293.8,458.1) | 1 (0,1) | 13.3 (6.3,23.5) | -96.3 (-98.4,-93.2) |
| United States of America | 28472 (27038,29822) | 118.9 (113.2,124.3) | 3013 (1349,5468) | 10.2 (4.6,18.7) | -91.4 (-96.1,-84.4) |
| Palestine | 3070 (2329,3936) | 773.8 (590.1,983.1) | 446 (203,880) | 79.7 (36.3,154.9) | -89.7 (-95.5,-78.9) |
| Syrian Arab Republic | 16062 (12115,22428) | 753.2 (569.3,1045.9) | 669 (264,1375) | 63.8 (25.1,132.6) | -91.5 (-96.6,-80.3) |
| United Arab Emirates | 832 (611,1130) | 392.8 (294.2,521.8) | 135 (62,252) | 36.8 (16.8,68.2) | -90.6 (-95.9,-82.4) |
| American Samoa | 41 (34,48) | 526.9 (445,622.6) | 1 (0,1) | 16.8 (8.3,28.6) | -96.8 (-98.5,-94.5) |
| Cook Islands | 26 (21,33) | 1262.7 (1007.3,1554.2) | 0 (0,1) | 24.9 (12.4,42.4) | -98 (-99.1,-96.4) |
| Guam | 61 (52,72) | 367.3 (316.3,424.6) | 2 (1,3) | 13.5 (6.3,23.1) | -96.3 (-98.2,-93.5) |
| Northern Mariana Islands | 17 (13,22) | 328 (253.8,420.3) | 0 (0,1) | 8.7 (4.2,15.3) | -97.4 (-98.7,-95.1) |
| Solomon Islands | 1225 (954,1572) | 2017.1 (1568.6,2571.4) | 70 (35,119) | 76.6 (38,129.6) | -96.2 (-98.1,-93.3) |
| Tokelau | 2 (1,2) | 894.6 (670.2,1139.1) | 0 (0,0) | 107.8 (51,189.2) | -87.9 (-94.5,-78.1) |
| Tuvalu | 44 (34,57) | 2786 (2150.8,3583.5) | 0 (0,1) | 36.2 (17.7,62.5) | -98.7 (-99.4,-97.6) |
| Lao People's Democratic Republic | 35841 (27191,45312) | 4665.8 (3548.7,5893.2) | 1749 (742,3359) | 212 (89.8,406.2) | -95.5 (-98.1,-90.9) |
| Malaysia | 9010 (7263,10826) | 412.4 (341.4,487.6) | 2452 (1019,4730) | 94.3 (39.3,181.6) | -77.1 (-90.3,-54.8) |
| Ireland | 546 (516,579) | 171.3 (161.2,182.1) | 27 (6,77) | 5.6 (1.2,15.9) | -96.7 (-99.3,-90.6) |
| Burkina Faso | 85599 (64582,107003) | 4219.7 (3174.8,5275.1) | 61302 (35533,93115) | 1450.3 (838.7,2197.6) | -65.6 (-79.3,-46.8) |
| People's Republic of China | 2231999 (1910836,2586118) | 2026.4 (1735.7,2347.4) | 43753 (24591,72959) | 67 (37.2,111.2) | -97.4 (-99.7,-91.2) |
| Kingdom of Cambodia | 104595 (87344,128606) | 985.2 (726.3,1274.1) | 313 (102,694) | 26.4 (12.5,44.4) | -99.6 (-99.9,-98.1) |
| Republic of Indonesia | 305999 (254377,376535) | 1511.6 (1243.3,1888) | 3950 (1516,8408) | 21.3 (5.7,49.1) | -92.8 (-96.8,-85.7) |
| Republic of Maldives | 421 (337,539) | 1028.2 (828.3,1311.3) | 4 (2,6) | 11.8 (5.9,20.5) | -67.1 (-81.3,-47.4) |
| Socialist Republic of Viet Nam | 139257 (114475,174417) | 2362.8 (2113.6,2650.6) | 1649 (445,3808) | 69.9 (26.8,134.9) | -71.5 (-86.2,-51.5) |
| Republic of Fiji | 510 (399,645) | 580.1 (457.7,727.7) | 27 (13,47) | 30.5 (14.9,53.7) | -99.2 (-99.7,-98.3) |
| Democratic Republic of Timor-Leste | 5983 (4897,7420) | 4361.3 (3359.4,5485) | 106 (52,182) | 72.1 (34.9,126.3) | -76.3 (-86.1,-62.8) |
| Republic of Kiribati | 120 (90,154) | 762.9 (612.6,934.9) | 4 (2,8) | 32.9 (16.1,55.5) | -92.2 (-98.1,-81.3) |
| Republic of the Philippines | 164784 (138550,198438) | 2211.1 (1870.8,2574.5) | 13070 (6003,25731) | 7.9 (2.9,18.2) | -80.6 (-89.3,-69.2) |
| Republic of the Union of Myanmar | 225304 (173606,282978) | 1778.8 (1499.5,2136.2) | 3712 (1795,6503) | 122 (56,239.4) | -72.7 (-83.6,-57.8) |
| Kingdom of Tonga | 114 (90,140) | 481.2 (407.1,566) | 5 (2,8) | 37.7 (9.5,88.1) | -93.4 (-97.4,-86.4) |
| Republic of Vanuatu | 256 (189,328) | 104.4 (83.4,136.1) | 11 (5,20) | 1.5 (0.7,2.4) | -78.6 (-93.3,-57.7) |
| Kingdom of Thailand | 32152 (25155,41411) | 201 (187.4,214.6) | 112 (18,320) | 3.5 (0.4,12.5) | -95.7 (-98.8,-90.2) |
| Independent State of Papua New Guinea | 22380 (17815,28312) | 363.9 (329.1,401.5) | 1876 (893,3463) | 2.8 (0.6,7.8) | -95.7 (-98.1,-92.3) |
| Republic of the Marshall Islands | 63 (50,78) | 4381.6 (3905.7,4906.5) | 2 (1,4) | 186.5 (55.7,426.4) | -99.1 (-99.8,-97.8) |
| Federated States of Micronesia | 190 (148,234) | 984.3 (747.1,1253) | 3 (1,5) | 29.6 (13.8,54.7) | -99.6 (-99.9,-99.2) |
| Republic of Uzbekistan | 138852 (127050,152556) | 931.6 (695.4,1187) | 9308 (4149,16340) | 27.3 (12.9,48.1) | -98.7 (-99.6,-97.3) |
| Independent State of Samoa | 246 (179,321) | 98.7 (91.4,106) | 7 (3,12) | 1.2 (0.2,3.5) | -94.8 (-98.2,-88.7) |
| Republic of Armenia | 8373 (7361,9577) | 5387.7 (4514.1,6601.1) | 888 (277,1735) | 18.5 (6,40.9) | -89.4 (-95.6,-79.1) |
| Republic of Azerbaijan | 62345 (53167,71831) | 923.4 (732.4,1126.9) | 1136 (242,2694) | 44.6 (20.8,75.9) | -71 (-84.4,-53.8) |
| Kyrgyz Republic | 27751 (24723,31080) | 975.8 (913.7,1051.1) | 1425 (426,3258) | 5.3 (2,11.3) | -94.4 (-98.3,-87.2) |
| Democratic Socialist Republic of Sri Lanka | 5364 (4629,6187) | 204.6 (190.4,218.9) | 56 (12,165) | 5.3 (0.6,17.6) | -98.6 (-99.3,-97.6) |
| Republic of Serbia | 2845 (2242,4004) | 320.3 (277.6,368.5) | 7 (3,15) | 3.2 (0.7,9.3) | -99.6 (-99.9,-98.8) |
| Republic of Albania | 8604 (7268,10025) | 4960.1 (4368.8,5648.4) | 12 (4,27) | 249 (108.5,476) | -77.9 (-86.8,-65.7) |
| Republic of Croatia | 727 (658,796) | 116.5 (108.5,124.2) | 6 (2,13) | 2.1 (0.6,4.9) | -88.6 (-94.9,-76.7) |
| Republic of Bulgaria | 5236 (4933,5618) | 88.9 (81.7,96.2) | 19 (7,41) | 1.6 (0.8,2.8) | -94.9 (-98.7,-87.2) |
| Czech Republic | 2227 (2066,2396) | 2281.3 (2005.9,2608.7) | 24 (9,50) | 488.3 (152.9,954.7) | -97 (-98.5,-94.3) |
| Republic of Slovenia | 425 (390,460) | 1384.2 (1150.8,1700.7) | 4 (1,11) | 18.4 (7.1,39.3) | -96.9 (-99.2,-91.8) |
| Slovak Republic | 2760 (2462,3076) | 224.9 (203,246.6) | 30 (9,72) | 77.9 (34.1,148.4) | -97.5 (-98.8,-95.7) |
| Republic of Poland | 5734 (5368,6103) | 418.2 (328.5,591.8) | 127 (14,440) | 1.2 (0.5,2.6) | -99.7 (-99.9,-99) |
| Republic of Kazakhstan | 42628 (38132,47824) | 643.7 (504.5,827.6) | 1376 (528,2658) | 2.5 (0.4,7.4) | -84.8 (-93.5,-70.7) |
| Republic of Tajikistan | 48221 (42464,54959) | 1287.4 (1009.7,1581.6) | 3353 (1462,6416) | 33 (16,56.7) | -97.9 (-99.3,-95.3) |
| Republic of Estonia | 412 (374,453) | 301.5 (276.6,327.5) | 3 (1,8) | 1.3 (0.1,6.6) | -73.6 (-86.9,-52.6) |
| Republic of Belarus | 3467 (2947,4063) | 3950.2 (3243.9,4888.7) | 258 (65,613) | 56.5 (27.6,97.2) | -63.7 (-77.2,-43.3) |
| Republic of Latvia | 582 (537,629) | 3269.5 (2603.5,4126.9) | 2 (0,8) | 117.5 (56,216.5) | -99.2 (-99.8,-97.9) |
| Republic of Lithuania | 641 (580,700) | 99.8 (94.9,104.4) | 149 (65,282) | 1.9 (1,3.3) | -99.7 (-99.9,-99.3) |
| Republic of Cyprus | 64 (50,84) | 234.7 (209.8,260.1) | 2 (1,3) | 2.1 (0.8,4.4) | -76.9 (-86.2,-63.6) |
| Republic of Finland | 581 (541,618) | 103.3 (73,147.9) | 11 (3,27) | 1.3 (0.7,2.3) | -90.9 (-98.2,-75.8) |
| Kingdom of Denmark | 457 (426,488) | 76.6 (72.1,81.2) | 10 (2,27) | 3.5 (1.3,7) | -94.7 (-97.4,-90.6) |
| Commonwealth of the Bahamas | 108 (90,132) | 321.6 (295.6,349.2) | 0 (0,2) | 2.6 (1,5.4) | -92 (-97.8,-81.2) |
| United Kingdom of Great Britain and Northern Ireland | 7933 (7510,8362) | 58.6 (54.4,62.5) | 360 (184,605) | 1.4 (0.7,2.3) | -99.1 (-99.7,-98) |
| Republic of Cuba | 2729 (2515,2920) | 674.1 (597.7,755.1) | 12 (4,58) | 8.9 (2.5,21.3) | -97.6 (-99.5,-94.1) |
| French Republic | 4224 (3937,4492) | 94.6 (89.2,100.4) | 293 (104,597) | 2.9 (0.7,7.8) | -99.1 (-99.8,-97.4) |
| Federal Republic of Germany | 5613 (5281,5918) | 7052.8 (6016.1,8124.3) | 64 (22,149) | 171.5 (36.5,406.8) | -81.6 (-92.7,-63.4) |
| State of Israel | 438 (402,475) | 110.5 (103.4,117.7) | 19 (9,31) | 8.8 (3.1,18.6) | -98.9 (-99.5,-97.9) |
| Republic of Italy | 3562 (3399,3733) | 320.3 (294.8,347.3) | 132 (67,220) | 11.1 (5.3,19.5) | -93.5 (-97.2,-89.1) |
| Grand Duchy of Luxembourg | 26 (24,28) | 4104.4 (3756.6,4507.7) | 1 (1,2) | 242.6 (108.2,426) | -94.5 (-97.4,-89.8) |
| Republic of Malta | 36 (33,40) | 86.5 (81.7,91.3) | 2 (1,4) | 0.5 (0.2,1.2) | -78.8 (-92.8,-57.5) |
| Kingdom of Spain | 2751 (2583,2912) | 77.8 (71.1,85.2) | 179 (47,480) | 1.9 (0.9,3.2) | -65.6 (-83.6,-37.7) |
| Kingdom of Sweden | 912 (846,976) | 382.7 (356.2,411.7) | 105 (29,241) | 0.7 (0.1,2.4) | -95.7 (-97.9,-91.9) |
| Swiss Confederation | 705 (654,757) | 334.8 (305.8,366.9) | 20 (10,33) | 2.5 (0.5,7.7) | -70.2 (-83.6,-49.4) |
| Eastern Republic of Uruguay | 891 (821,965) | 90.4 (85.2,96) | 29 (14,51) | 7.2 (2,16.8) | -98.6 (-99.3,-97.3) |
| Commonwealth of Dominica | 24 (19,29) | 115.8 (104.6,128.6) | 0 (0,0) | 4.1 (2,7) | -83.2 (-93.3,-63.7) |
| Republic of Singapore | 864 (803,929) | 129.1 (117.5,142.4) | 4 (0,13) | 47.3 (23.6,72.3) | -63.4 (-82.1,-43.7) |
| Hellenic Republic | 611 (573,647) | 94.2 (87.1,100.7) | 4 (1,12) | 3.2 (0.5,9.7) | -94.1 (-97.4,-89.1) |
| Kingdom of Norway | 532 (488,573) | 428.4 (357.6,517.7) | 11 (2,33) | 1.7 (0.5,8) | -95.2 (-97.8,-91.3) |
| Portuguese Republic | 1421 (1334,1506) | 105.4 (99,112.4) | 81 (10,270) | 1.3 (0.2,4) | -82.1 (-89.9,-70.4) |
| Republic of Trinidad and Tobago | 398 (339,461) | 77 (72.1,82.2) | 1 (0,5) | 0.3 (0,0.8) | -96.7 (-98.2,-94.3) |
| Republic of Costa Rica | 1407 (1289,1542) | 605.7 (507.7,710.9) | 15 (5,31) | 3.1 (1,15.4) | -59.3 (-90.5,-3.9) |
| Kingdom of the Netherlands | 1192 (1107,1275) | 113.8 (106,122.9) | 64 (10,197) | 1.8 (0.9,3.1) | -98.7 (-99.8,-96.5) |
| Republic of Iceland | 33 (30,36) | 305.7 (281.3,327.7) | 19 (10,29) | 1.4 (0.4,6.7) | -74.5 (-84.3,-59.3) |
| Republic of Chile | 7179 (6721,7602) | 137.9 (131.8,144.4) | 67 (19,147) | 4.5 (2.3,7.6) | -84.7 (-93.1,-72.6) |
| Republic of Haiti | 36103 (28615,45382) | 333.2 (311.3,357.4) | 120 (33,601) | 22 (8.8,45.7) | -94 (-97.3,-88.3) |
| Republic of Guatemala | 26663 (23994,29766) | 280 (225.3,341.6) | 4474 (1691,9065) | 2.8 (0.8,13.9) | -80.3 (-92.4,-60.1) |
| Argentine Republic | 11056 (10327,11861) | 3154.8 (2513.2,3935.4) | 823 (334,1677) | 7.5 (2.1,37.8) | -94.6 (-97.8,-88.5) |
| Kingdom of Belgium | 973 (911,1035) | 880.1 (729.1,1036.2) | 128 (45,267) | 8.1 (1.6,21.2) | -98.8 (-99.4,-97.6) |
| Republic of Peru | 74240 (63584,87237) | 3245.9 (2344,4134) | 15003 (4612,26755) | 28.2 (6.4,68.6) | -80.6 (-91,-67.8) |
| Republic of El Salvador | 6997 (5779,8252) | 514.4 (483.3,543.2) | 47 (9,124) | 4.6 (1.3,10.2) | -91.5 (-96.4,-83) |
| Plurinational State of Bolivia | 33780 (24290,43219) | 3672.8 (3034.1,4533.9) | 328 (75,798) | 205.6 (67.5,438) | -73.7 (-84.8,-58.6) |
| Republic of Ecuador | 13685 (12251,15199) | 1778.3 (1607.5,1978.7) | 1443 (285,3868) | 308.9 (116.7,625.8) | -83.4 (-93.6,-64.6) |
| Principality of Andorra | 3 (2,5) | 1956.9 (1495.9,2495.7) | 0 (0,0) | 108.9 (43.4,214.7) | -76.2 (-85.2,-61.9) |
| Republic of Austria | 389 (363,413) | 1729.7 (1310.7,2328.2) | 15 (8,26) | 59.3 (27.1,114.6) | -99.7 (-99.9,-99.2) |
| Republic of Guyana | 708 (590,835) | 1115.8 (992.4,1245.7) | 2 (1,11) | 66.9 (18.8,152.5) | -98.3 (-99.2,-97.1) |
| Republic of Suriname | 272 (214,329) | 995.3 (893.8,1102.6) | 1 (0,5) | 90.8 (17.9,243) | -82.6 (-93.3,-65.7) |
| Republic of Colombia | 31634 (27375,36151) | 1120.3 (929.7,1329.3) | 1194 (266,3026) | 155.5 (44.5,362.2) | -76.9 (-87.8,-58.9) |
| Republic of Panama | 1284 (1090,1488) | 970.9 (801.4,1279.7) | 664 (149,1576) | 63 (29.6,99.4) | -99.6 (-100,-97.8) |
| United Mexican States | 141400 (128181,158640) | 2494.4 (2141.7,2924.2) | 5936 (3035,10170) | 453.8 (139.4,810.5) | -97.4 (-98.8,-95.3) |
| Republic of Honduras | 9333 (7712,11117) | 1194 (1084,1338.6) | 1621 (464,3765) | 61.4 (31.5,104.9) | -74.5 (-84.4,-61.3) |
| Republic of Nicaragua | 8809 (7489,10313) | 1038 (819.1,1315.8) | 431 (148,887) | 97.3 (42.2,182.9) | -90.6 (-95.9,-80.7) |
| Federative Republic of Brazil | 171673 (152464,191924) | 647.9 (510.6,811.4) | 12160 (3403,27554) | 33.4 (8.1,77.4) | -98.7 (-99.6,-96.9) |
| Republic of Paraguay | 3925 (3080,4936) | 2126.1 (1693.5,2550.9) | 211 (51,489) | 634.6 (236.8,1171.8) | -77.7 (-91.5,-55.4) |
| Bolivarian Republic of Venezuela | 13429 (12492,14407) | 742.8 (545.1,1116.5) | 1182 (618,2005) | 81.8 (31.8,164.6) | -94.4 (-97.7,-88.9) |
| People's Democratic Republic of Algeria | 30774 (22429,42452) | 712.6 (591.8,852.9) | 3091 (1405,5748) | 78.3 (21.3,184.1) | -81.1 (-89,-70.4) |
| Arab Republic of Egypt | 323648 (267038,399910) | 2177.2 (1655.9,3064.3) | 24907 (8185,53369) | 184.6 (82.4,343.6) | -98.6 (-99.6,-96.5) |
| Islamic Republic of Iran | 73868 (60793,97872) | 277.3 (209,353.9) | 3935 (1889,6038) | 5.9 (2,12.8) | -96.4 (-98.2,-93.8) |
| Kingdom of Morocco | 72441 (55276,92458) | 740.1 (642.1,844.1) | 3412 (1363,6712) | 33.6 (7.4,84.8) | -81.8 (-94.6,-67) |
| Republic of Iraq | 33386 (26289,42467) | 1326.8 (1131,1553.4) | 3935 (1710,7407) | 69.4 (23.8,142.6) | -95 (-97.8,-90.4) |
| Hashemite Kingdom of Jordan | 4351 (3589,5227) | 1411.3 (981.2,1931.6) | 812 (222,1913) | 401.7 (187.4,646.1) | -81.5 (-88.1,-71.9) |
| Kingdom of Bahrain | 239 (202,295) | 1641.2 (1208.8,2134.6) | 34 (15,68) | 432 (257.5,667.6) | -97.1 (-98.7,-94.7) |
| Lebanese Republic | 3611 (2537,4899) | 631.9 (500.2,762.9) | 438 (202,829) | 2.2 (0.6,11.3) | -71.7 (-86.4,-52.6) |
| Kingdom of Saudi Arabia | 16297 (12431,22055) | 854.6 (629,1172.5) | 1028 (444,1974) | 71 (32.3,132.3) | -98.4 (-99.2,-97.3) |
| Republic of Turkey | 120682 (91267,162782) | 2200.3 (1626.3,3224.5) | 3353 (1551,6476) | 543.9 (335.4,839.2) | -70.5 (-88.5,-41.9) |
| State of Libya | 3058 (2283,4127) | 462.4 (393.9,534.6) | 363 (150,721) | 188.3 (42.2,446.3) | -77.1 (-88.8,-58.4) |
| Republic of Tunisia | 13214 (9015,21173) | 368.2 (338.7,402.3) | 557 (232,1084) | 4.8 (1.5,10.1) | -99.7 (-99.9,-98.3) |
| State of Kuwait | 745 (654,846) | 4158.1 (3374,5033.4) | 330 (152,625) | 426 (155.7,869.2) | -70.2 (-89,-45.7) |
| State of Qatar | 138 (102,177) | 348.7 (298.5,402.3) | 10 (4,23) | 1.3 (0.4,6.2) | -57.5 (-76.1,-30.3) |
| Republic of Yemen | 64157 (48488,90594) | 432.1 (381,490.1) | 8358 (3737,15544) | 128.9 (59.2,243.2) | -90.1 (-94.7,-83) |
| Republic of Equatorial Guinea | 2275 (1593,3036) | 2224.9 (1690.8,2821.2) | 629 (263,1142) | 793.9 (492.8,1220.9) | -99.8 (-100,-99.4) |
| Federal Democratic Republic of Nepal | 148205 (120484,179046) | 2544.1 (1788.2,3381.2) | 13053 (4762,26676) | 389.8 (166.2,694) | -91.7 (-96.5,-83.1) |
| Islamic Republic of Pakistan | 422213 (333746,522095) | 501.1 (375.9,671.5) | 209316 (115779,336797) | 84.4 (34.7,169.9) | -98.7 (-99.5,-97.3) |
| Islamic Republic of Afghanistan | 69646 (53087,92776) | 2168.3 (1719.4,2671.2) | 14229 (6214,28315) | 712.4 (393.8,1146.3) | -96.5 (-98.3,-93.9) |
| Republic of India | 2487129 (1976069,2987872) | 531.8 (495.8,569.8) | 674441 (251584,1245583) | 52.7 (27.6,89.6) | -98.2 (-99.5,-95.9) |
| Republic of Djibouti | 1123 (821,1471) | 704.7 (540.4,942.2) | 594 (352,924) | 38.1 (16.7,72.3) | -86.1 (-95.9,-66.6) |
| State of Eritrea | 14220 (10799,18250) | 901.8 (639,1219.5) | 6897 (4256,10717) | 102.7 (47,196.4) | -99.5 (-99.8,-97.6) |
| Sultanate of Oman | 2418 (1770,3705) | 3129.6 (2322.8,4052) | 306 (119,620) | 607.2 (363.4,929.4) | -93.1 (-96.7,-86.4) |
| Kingdom of Bhutan | 2270 (1525,3059) | 3575.8 (2745.3,4764) | 223 (94,423) | 257.1 (113.1,508.2) | -64.3 (-80.1,-43.8) |
| Federal Democratic Republic of Ethiopia | 309971 (232530,391464) | 3024.5 (2278.6,3810.9) | 74705 (48180,113221) | 483.3 (313.3,726.3) | -66.2 (-79.6,-44.3) |
| Gabonese Republic | 2308 (1614,3144) | 1267.6 (867.2,2024.4) | 784 (348,1372) | 63.4 (26.2,124.1) | -83 (-89.9,-73.3) |
| Republic of Madagascar | 71592 (61451,83189) | 608.1 (523,699.5) | 25398 (8842,50138) | 32.5 (16.5,56.1) | -97 (-98.9,-94.1) |
| Republic of Rwanda | 43359 (31976,56058) | 399.3 (341.5,489.2) | 10238 (6053,15727) | 38.8 (16.4,76) | -96.4 (-98.3,-93.7) |
| Republic of Mozambique | 57385 (42246,84514) | 1112.7 (875.5,1396.3) | 26516 (16345,41267) | 219.3 (83.5,433.2) | -96.6 (-99.5,-90) |
| Republic of Seychelles | 46 (40,54) | 1952.2 (1615.4,2505.9) | 3 (1,5) | 533.5 (327.4,799.8) | -99.5 (-99.8,-98.9) |
| People's Republic of Bangladesh | 586056 (491258,692060) | 1443.6 (1019.2,1948.5) | 62391 (26978,116637) | 409 (183,710.7) | -89 (-96.9,-73.9) |
| Republic of Burundi | 26858 (19324,35220) | 3072.9 (2336.7,3948.7) | 11659 (6455,19738) | 596.6 (270.6,979.1) | -65.4 (-85.1,-34.6) |
| Union of the Comoros | 2610 (1994,3410) | 2952.5 (2064.5,3960.5) | 538 (336,802) | 703.9 (434.4,1135) | -83.5 (-93.5,-66.8) |
| Republic of Kenya | 86975 (71633,111968) | 3000 (2520.4,3534.2) | 29027 (17745,43881) | 456.2 (197,853.5) | -95.3 (-97.7,-91.9) |
| Republic of Uganda | 55522 (38575,79641) | 2380.9 (1723,3079.8) | 29486 (10685,63387) | 565.3 (318.4,940.8) | -71 (-82.5,-56) |
| Republic of Zambia | 47959 (37730,61528) | 3061.5 (2637.7,3546.1) | 23302 (12903,35654) | 649 (225.7,1280.7) | -99.5 (-99.9,-97.9) |
| Republic of Angola | 65088 (49102,84323) | 2395 (1910.9,3001.8) | 31339 (13972,52095) | 528.1 (339.1,780.1) | -97.7 (-98.8,-96.1) |
| Republic of the Congo | 5657 (3850,7863) | 5657.9 (4198,7422) | 2278 (1053,3701) | 1047 (607.2,1606.4) | -99.2 (-99.8,-97.8) |
| Republic of Mauritius | 478 (438,520) | 1499.2 (1238.8,1837.3) | 15 (8,27) | 442.3 (165.2,833.1) | -63 (-77.1,-45) |
| Federal Republic of Somalia | 49275 (34112,66728) | 2233.6 (1485.6,3011.2) | 29483 (17869,47629) | 369.9 (156.6,701.3) | -89.8 (-96.3,-78.5) |
| Kingdom of Eswatini | 2030 (1631,2530) | 1606.1 (1249.7,2044) | 351 (142,691) | 478.2 (257.6,783.3) | -95 (-97.9,-89.4) |
| Republic of Zimbabwe | 28869 (24560,33889) | 3039.1 (2404.3,3767.2) | 7839 (3075,15448) | 882.4 (503.8,1356.9) | -71 (-82.8,-55.3) |
| Kingdom of Lesotho | 3651 (2993,4493) | 4945.9 (3760.8,6313.6) | 871 (326,1640) | 932.3 (531.7,1417.4) | -70.3 (-88.8,-38.3) |
| Republic of Namibia | 2327 (1797,2923) | 4649 (3769.2,5544) | 453 (170,921) | 1348 (768.3,2069.2) | -95.5 (-98.3,-90.9) |
| Republic of Botswana | 2227 (1713,2790) | 1049.8 (821.9,1318.1) | 504 (192,996) | 172.8 (64.6,352.2) | -87.8 (-92.5,-80.8) |
| Republic of South Africa | 87963 (74911,105683) | 2381.7 (1834.5,3110.5) | 9425 (3764,19129) | 820.3 (374.8,1419) | -97.3 (-98.7,-95) |
| Republic of Malawi | 63763 (46286,84955) | 3060.1 (2242.5,4031.1) | 20323 (11588,31155) | 781.8 (451.8,1196) | -70.2 (-86.2,-41.7) |
| Republic of the Gambia | 4657 (3702,5857) | 2343.2 (1865.3,2814.5) | 1755 (1116,2595) | 316 (181.1,543.6) | -94.7 (-97.6,-90.2) |
| Republic of Benin | 31337 (24624,38758) | 1823.4 (1560.3,2182.8) | 20604 (11817,31720) | 194 (77.4,393.5) | -99 (-99.8,-97.1) |
| Republic of Liberia | 19245 (14459,25358) | 453.6 (417.1,493.3) | 3496 (2039,5558) | 21.1 (10.5,36.3) | -94.9 (-97.3,-91) |
| Republic of the Niger | 101553 (76222,132982) | 2932.7 (2228.5,3814) | 54213 (31536,82902) | 676.8 (423.2,1006.7) | -84 (-90.3,-75.4) |
| Republic of Chad | 45330 (35822,56017) | 844.7 (634.9,1121.3) | 46455 (29176,69975) | 195.4 (115.1,330.2) | -96.7 (-98.4,-94.5) |
| Republic of Cte d'Ivoire | 71504 (54851,94995) | 2925.6 (2291.7,3745.1) | 24357 (12461,40327) | 849.5 (474.4,1295.5) | -75.3 (-85.2,-57.7) |
| Republic of Guinea-Bissau | 5416 (4111,6911) | 2800.2 (2138.2,3555.5) | 1444 (880,2195) | 474.6 (294.8,715.7) | -86.5 (-92.4,-76.2) |
| Republic of Cameroon | 49930 (38297,65241) | 1443.6 (1016.4,2092) | 38721 (17661,67201) | 428.5 (157.3,910.2) | -89 (-96.1,-77.2) |
| Republic of Guinea | 61822 (47081,78635) | 2294.3 (1853.4,2764.9) | 20812 (11754,31806) | 604.8 (304.5,1078.1) | -99.6 (-99.9,-98.3) |
| Republic of Cabo Verde | 485 (356,645) | 1426.2 (1151.3,1770.8) | 85 (51,144) | 262 (107.3,516.4) | -96.6 (-98.6,-93) |
| Democratic Republic of Sao Tome and Principe | 484 (384,583) | 3929.5 (2926,5187.1) | 69 (39,121) | 478.1 (279.7,752.7) | -80.6 (-89.5,-67.2) |
| Republic of Mali | 36781 (28890,46838) | 4205 (3079.7,5571.6) | 33346 (19853,49754) | 754.1 (429.9,1253.9) | -94.1 (-97.4,-89.4) |
| Islamic Republic of Mauritania | 7293 (5815,8848) | 1965.6 (1529.2,2514.3) | 2966 (1857,4639) | 713.8 (429.2,1063.9) | -98.2 (-99.1,-96.8) |
| Federal Republic of Nigeria | 805605 (657267,956300) | 1658.9 (1417.4,1940) | 507757 (290920,780237) | 369.4 (145.9,726.4) | -99.8 (-99.9,-98.8) |
| Republic of Ghana | 42601 (32848,54702) | 3148.5 (2417.5,4149.4) | 20663 (11086,34485) | 1064.9 (620.9,1879) | -94 (-98.3,-85.7) |
| Republic of Senegal | 34928 (28134,41987) | 105 (77.3,140.6) | 13363 (6666,24067) | 5.8 (2.8,9.8) | -99.4 (-99.8,-98.6) |
| Republic of Sierra Leone | 35893 (26447,47678) | 2907.6 (2246.8,3864.7) | 10022 (5684,16724) | 564.7 (288.5,934.8) | -90.3 (-96,-80) |
| Togolese Republic | 17067 (13376,21439) | 83.7 (65.3,110.4) | 11377 (6149,18054) | 1.2 (0.6,2.2) | -95.5 (-98.9,-88) |
| Republic of San Marino | 1 (1,2) | 3351.4 (2640.3,4116.9) | 0 (0,0) | 1238.5 (778.7,1857.9) | -92 (-97.2,-82.9) |
| Republic of South Sudan | 34748 (26646,45975) | 2422.6 (1907.4,3037.4) | 17570 (10189,31389) | 1028.7 (560.7,1618.5) | -99.1 (-99.7,-98.1) |
| Republic of Nauru | 22 (17,27) | 1364.6 (1083.7,1683.2) | 1 (0,2) | 80.2 (36.6,141.2) | -98.3 (-99.8,-93.7) |
| Republic of Niue | 2 (2,3) | 1893.9 (1520.3,2287.4) | 0 (0,0) | 482.5 (304.9,746.4) | -98.6 (-99.4,-97.3) |
| Principality of Monaco | 3 (3,4) | 938 (746.1,1162.5) | 0 (0,0) | 215.1 (110.3,356.5) | -99 (-99.7,-95.1) |
| Republic of Sudan | 99994 (73431,136865) | 2656.4 (1942.6,3657.6) | 8607 (3780,16177) | 158.8 (69.7,297.9) | -98.8 (-99.8,-96.2) |
| Republic of Palau | 17 (13,22) | 1167.2 (867.6,1508.3) | 0 (0,1) | 50.5 (23.6,86.4) | -98.1 (-99,-96.6) |

| Table S2. Deaths of RSV-related LRIs in 1990 and 2021 and the percentage change in the age-standardised rates (ASRs) per 1000, by location | | | | | |
| --- | --- | --- | --- | --- | --- |
|  | 1990 | | 2021 | | Percentage change in the |
|  | No (95% UI) | ASRs per 1000 (95% UI) | No (95% UI) | ASRs per 1000 (95% UI) | ASRs per 1000 |
| Australia | 29 (26,31) | 1.9 (1.7,2) | 0 (0,1) | 0 (0,0) | -99.5 (-99.9,-98.8) |
| New Zealand | 14 (13,16) | 4.5 (4.1,4.9) | 0 (0,0) | 0 (0,0) | -99.7 (-99.9,-99.2) |
| Antigua and Barbuda | 0 (0,0) | 4.4 (3.8,4.9) | 0 (0,0) | 0 (0,0.2) | -99.3 (-99.8,-96.8) |
| Barbados | 1 (1,1) | 4.3 (3.8,4.7) | 0 (0,0) | 0 (0,0.1) | -99.4 (-99.8,-97) |
| Belize | 3 (2,3) | 10.2 (9.2,11.4) | 0 (0,0) | 0 (0,0.2) | -99.7 (-99.9,-98.4) |
| Bermuda | 0 (0,0) | 2.8 (2.5,3.1) | 0 (0,0) | 0 (0,0) | -99.7 (-99.9,-98.5) |
| Dominican Republic | 88 (72,105) | 9.2 (7.6,10.8) | 0 (0,1) | 0 (0,0.1) | -99.8 (-99.9,-99) |
| Grenada | 1 (1,1) | 7.8 (6.7,9) | 0 (0,0) | 0 (0,0.2) | -99.5 (-99.9,-97.8) |
| Jamaica | 9 (8,10) | 3.7 (3.4,4.1) | 0 (0,0) | 0 (0,0.1) | -99.6 (-99.9,-98.3) |
| Puerto Rico | 15 (14,16) | 4.8 (4.5,5.1) | 0 (0,0) | 0 (0,0.1) | -99.7 (-99.9,-98.7) |
| Saint Kitts and Nevis | 0 (0,0) | 8.1 (7.4,8.8) | 0 (0,0) | 0 (0,0.2) | -99.5 (-99.8,-97.5) |
| Saint Lucia | 1 (1,1) | 5.1 (4.5,5.8) | 0 (0,0) | 0 (0,0.1) | -99.5 (-99.9,-97.6) |
| Saint Vincent and the Grenadines | 1 (0,1) | 5.6 (4.8,6.5) | 0 (0,0) | 0 (0,0.1) | -99.5 (-99.9,-97.8) |
| United States Virgin Islands | 0 (0,0) | 3.1 (2.6,3.7) | 0 (0,0) | 0 (0,0) | -99.8 (-99.9,-98.8) |
| Georgia | 147 (128,167) | 34.1 (29.7,39.1) | 0 (0,1) | 0.1 (0,0.2) | -99.8 (-100,-99.3) |
| Mongolia | 181 (149,213) | 53.5 (44.1,63) | 1 (0,4) | 0.3 (0,1.2) | -99.5 (-100,-97.8) |
| Turkmenistan | 331 (292,381) | 56.1 (49.6,64.5) | 17 (7,30) | 3.2 (1.4,5.7) | -94.4 (-97.3,-89.6) |
| Bosnia and Herzegovina | 8 (7,9) | 2.3 (2,2.8) | 0 (0,0) | 0 (0,0) | -99.2 (-99.7,-98.1) |
| Hungary | 23 (21,24) | 3 (2.8,3.3) | 0 (0,1) | 0 (0,0.1) | -99.2 (-99.7,-98.3) |
| Montenegro | 2 (1,2) | 3.5 (2.8,4.5) | 0 (0,0) | 0 (0,0) | -99.4 (-99.8,-98.7) |
| North Macedonia | 15 (13,19) | 9.3 (7.6,11.5) | 0 (0,0) | 0 (0,0) | -99.8 (-99.9,-99.5) |
| Romania | 374 (351,394) | 23.2 (21.7,24.5) | 1 (0,4) | 0.1 (0,0.2) | -99.7 (-100,-99) |
| Central African Republic | 241 (164,325) | 46.1 (32.8,61) | 136 (63,228) | 17.8 (8.6,29) | -61.3 (-81.4,-35.5) |
| Democratic Republic of the Congo | 2363 (1609,3158) | 32.4 (23,42.1) | 983 (467,1606) | 9.8 (4.8,15.4) | -69.7 (-84.3,-51.5) |
| Democratic People's Republic of Korea | 363 (275,473) | 15.3 (11.8,19.8) | 19 (10,33) | 1 (0.5,1.8) | -93.4 (-96.8,-87.6) |
| Taiwan (Province of China) | 79 (75,84) | 5.7 (5.3,6) | 33 (18,54) | 1.1 (0.6,1.8) | -81.3 (-89.8,-69.1) |
| Republic of Moldova | 62 (55,71) | 15.8 (13.9,18.1) | 2 (1,4) | 0.9 (0.3,1.8) | -94.5 (-98.2,-88.6) |
| Russian Federation | 760 (728,793) | 7.2 (6.9,7.5) | 50 (13,114) | 0.4 (0.1,0.9) | -94.7 (-98.7,-87.8) |
| Ukraine | 189 (166,215) | 5.3 (4.6,6.1) | 8 (4,13) | 0.2 (0.1,0.4) | -95.6 (-97.9,-92.8) |
| United Republic of Tanzania | 2122 (1669,2599) | 42.8 (33.9,51.9) | 906 (509,1479) | 11.6 (6.7,18.7) | -72.8 (-84,-56.6) |
| Brunei Darussalam | 1 (1,1) | 4.5 (4,5.2) | 0 (0,0) | 0 (0,0) | -99.9 (-100,-99.4) |
| Japan | 646 (580,706) | 4.8 (4.3,5.2) | 2 (0,12) | 0 (0,0) | -99.9 (-100,-99.5) |
| Republic of Korea | 93 (79,110) | 3.6 (3,4.3) | 0 (0,0) | 0 (0,0) | -100 (-100,-100) |
| Canada | 92 (82,99) | 3.3 (2.9,3.5) | 7 (2,18) | 0.1 (0,0.3) | -96.8 (-99.2,-92.2) |
| Greenland | 0 (0,0) | 6.1 (5,7.3) | 0 (0,0) | 0.3 (0.1,0.4) | -95.8 (-98.1,-92.6) |
| United States of America | 876 (787,944) | 3 (2.7,3.2) | 107 (48,193) | 0.2 (0.1,0.4) | -92.3 (-96.6,-85.9) |
| Palestine | 35 (27,45) | 9.9 (7.9,12.2) | 6 (3,11) | 1.2 (0.6,2.3) | -87.6 (-94.4,-75.7) |
| Syrian Arab Republic | 183 (138,255) | 9.1 (7,12.3) | 11 (5,21) | 1.1 (0.4,2.1) | -88.2 (-95,-74.5) |
| United Arab Emirates | 10 (7,13) | 6.2 (5,7.8) | 2 (1,4) | 0.9 (0.4,1.6) | -85.7 (-93.4,-73.5) |
| American Samoa | 0 (0,1) | 7.5 (6.5,8.6) | 0 (0,0) | 0.3 (0.1,0.4) | -96.6 (-98.3,-94.1) |
| Cook Islands | 0 (0,0) | 17.8 (14.8,21.1) | 0 (0,0) | 0.4 (0.2,0.7) | -97.6 (-98.9,-95.9) |
| Guam | 1 (1,1) | 5.7 (5,6.4) | 0 (0,0) | 0.2 (0.1,0.3) | -97 (-98.5,-94.7) |
| Northern Mariana Islands | 0 (0,0) | 5.8 (4.7,7.1) | 0 (0,0) | 0.2 (0.1,0.3) | -97.1 (-98.6,-94.8) |
| Solomon Islands | 14 (11,18) | 27 (21.2,33.6) | 1 (0,1) | 1.1 (0.5,1.8) | -96 (-97.9,-93) |
| Tokelau | 0 (0,0) | 11.9 (9.1,14.8) | 0 (0,0) | 1.3 (0.6,2.3) | -89.1 (-95,-80.6) |
| Tuvalu | 1 (0,1) | 33.2 (26.1,42.3) | 0 (0,0) | 0.5 (0.3,0.9) | -98.5 (-99.2,-97.2) |
| Lao People's Democratic Republic | 404 (307,510) | 53.9 (41.3,67.7) | 21 (9,39) | 2.7 (1.1,5.1) | -95.1 (-97.9,-90) |
| Malaysia | 118 (98,138) | 6.6 (5.6,7.7) | 68 (30,135) | 2.8 (1.2,5.6) | -57.4 (-81.7,-15.4) |
| Ireland | 20 (19,22) | 5.9 (5.3,6.3) | 1 (0,4) | 0.2 (0,0.5) | -96.8 (-99.3,-90.8) |
| Burkina Faso | 969 (733,1210) | 50.2 (38.3,62) | 700 (405,1060) | 17.9 (10.4,26.8) | -64.3 (-78.4,-44.8) |
| Republic of Indonesia | 3471 (2883,4260) | 16.1 (13.4,19.6) | 50 (19,106) | 0.2 (0.1,0.5) | -96.9 (-98.4,-94.4) |
| Kingdom of Cambodia | 1179 (986,1448) | 17.8 (14.8,22) | 4 (1,8) | 0.3 (0.1,0.7) | -75.9 (-92.6,-52.2) |
| Socialist Republic of Viet Nam | 1586 (1305,1975) | 23.9 (20.6,27.5) | 23 (7,53) | 1 (0.6,1.6) | -95.6 (-98.7,-89.8) |
| Republic of Azerbaijan | 698 (596,804) | 63 (53.2,76.9) | 13 (3,31) | 0.2 (0.1,0.5) | -99.6 (-99.9,-98) |
| Republic of Poland | 92 (87,96) | 12.7 (10.5,15.9) | 5 (1,17) | 0.2 (0.1,0.3) | -96.8 (-98.4,-94) |
| People's Republic of China | 25508 (21897,29533) | 50.2 (38.8,62.7) | 857 (484,1432) | 0.9 (0.4,1.5) | -91.1 (-97.6,-79.2) |
| Republic of Maldives | 5 (4,6) | 13 (9.9,16.2) | 0 (0,0) | 0.4 (0.2,0.7) | -95.6 (-97.8,-92.5) |
| Republic of the Philippines | 1893 (1598,2273) | 2 (1.8,2.1) | 181 (83,347) | 0 (0,0) | -98.8 (-99.7,-97.3) |
| Republic of the Union of Myanmar | 2549 (1966,3197) | 22 (19,26) | 44 (21,76) | 1.9 (0.9,3.7) | -99.4 (-99.9,-98.2) |
| Federal Republic of Germany | 199 (180,216) | 26.8 (24,30) | 4 (1,9) | 1 (0.4,1.9) | -98.6 (-99.6,-96.7) |
| Independent State of Samoa | 3 (2,4) | 7.8 (6.4,9.6) | 0 (0,0) | 0.4 (0.2,0.7) | -94.5 (-98.1,-88.1) |
| Democratic Socialist Republic of Sri Lanka | 71 (62,81) | 26.3 (22.4,30.4) | 1 (0,4) | 0.1 (0,0.2) | -98.6 (-99.6,-97.1) |
| Kyrgyz Republic | 311 (278,349) | 10.4 (8.7,12.4) | 16 (5,38) | 0.5 (0.2,0.8) | -95 (-97.9,-89.6) |
| Republic of Kazakhstan | 481 (431,540) | 5.6 (4.8,6.5) | 18 (7,36) | 0.6 (0.2,1.4) | -93.3 (-98.3,-83.6) |
| Kingdom of Thailand | 382 (299,484) | 4.4 (4,4.8) | 4 (1,11) | 0 (0,0.1) | -97.8 (-98.9,-95.8) |
| Democratic Republic of Timor-Leste | 67 (55,84) | 2.9 (2.8,3.1) | 1 (1,2) | 0.1 (0,0.3) | -93.2 (-97.9,-84.5) |
| Republic of Fiji | 6 (5,8) | 5.3 (5,5.6) | 0 (0,1) | 0 (0,0.2) | -99.5 (-100,-97.3) |
| Kingdom of Tonga | 1 (1,2) | 49.4 (44.1,55.3) | 0 (0,0) | 2.2 (0.7,5) | -63.7 (-81.8,-42.1) |
| Republic of Vanuatu | 3 (2,4) | 11.9 (9.2,14.9) | 0 (0,0) | 0.4 (0.2,0.7) | -95.6 (-98.8,-87.9) |
| Republic of Armenia | 94 (83,108) | 45.7 (37.7,56.2) | 13 (4,26) | 0.7 (0.4,1.2) | -66.5 (-80.9,-46.5) |
| Republic of Kiribati | 1 (1,2) | 56.5 (49.9,64.3) | 0 (0,0) | 2.9 (1.3,5.5) | -92.3 (-96.5,-84.8) |
| French Republic | 189 (168,207) | 4.6 (4.3,4.9) | 19 (7,39) | 0.1 (0,0.1) | -97.5 (-99.5,-94) |
| Republic of Tajikistan | 543 (478,619) | 13 (12.3,13.9) | 38 (17,73) | 0.1 (0,0.2) | -73.1 (-83.5,-59.2) |
| Independent State of Papua New Guinea | 251 (200,318) | 79.2 (67.6,91.2) | 21 (10,39) | 2 (0.4,4.6) | -83.7 (-92.5,-68) |
| Argentine Republic | 155 (146,165) | 25.8 (22.7,29.4) | 29 (11,58) | 6.2 (1.9,12.1) | -89.4 (-97.9,-71.3) |
| Republic of Croatia | 13 (12,14) | 5 (4.4,5.6) | 0 (0,0) | 0.1 (0,0.2) | -78.2 (-87.5,-66) |
| Republic of Cuba | 47 (44,50) | 3.5 (3.3,3.8) | 0 (0,2) | 0 (0,0.1) | -98.1 (-99.7,-94.9) |
| Federated States of Micronesia | 2 (2,3) | 37.7 (30.5,47.4) | 0 (0,0) | 1.4 (0.6,2.5) | -98.5 (-99.3,-97.4) |
| Republic of Bulgaria | 77 (73,81) | 16.3 (12.8,19.8) | 0 (0,1) | 0.5 (0.2,0.8) | -98.4 (-99.2,-97.1) |
| Republic of Uzbekistan | 1558 (1426,1712) | 12 (9.3,15.1) | 105 (47,185) | 0.4 (0.2,0.6) | -98.8 (-99.6,-97.2) |
| Republic of the Marshall Islands | 1 (1,1) | 46.3 (42.4,50.8) | 0 (0,0) | 2.8 (1.2,4.9) | -95.2 (-97.7,-91.6) |
| Republic of Albania | 99 (84,115) | 8 (6.4,10.1) | 0 (0,0) | 0.1 (0,0.1) | -67.7 (-80.5,-50) |
| Czech Republic | 38 (36,41) | 2.5 (2.2,2.7) | 1 (0,2) | 0.1 (0,0.2) | -99.4 (-99.8,-98.8) |
| Slovak Republic | 44 (39,48) | 12.2 (9.9,14.5) | 1 (0,2) | 0.6 (0.3,1) | -98.2 (-99.1,-96.9) |
| Republic of Peru | 859 (740,1006) | 5.2 (4.2,7.1) | 259 (83,439) | 0 (0,0.1) | -99.1 (-99.8,-97.4) |
| Republic of Slovenia | 8 (7,9) | 9.8 (8.8,10.8) | 0 (0,0) | 0.1 (0,0.3) | -88.1 (-95.1,-76.7) |
| Republic of Belarus | 41 (36,48) | 3.7 (3.4,4) | 5 (1,13) | 0 (0,0.1) | -66.8 (-81.9,-47.3) |
| Republic of Estonia | 5 (5,6) | 5.1 (4.7,5.5) | 0 (0,0) | 0 (0,0.1) | -95.9 (-97.7,-93) |
| Republic of Serbia | 36 (29,49) | 2.7 (2.5,3) | 0 (0,1) | 1.2 (0.5,2.4) | -54.2 (-80.2,-13.3) |
| Republic of Latvia | 8 (7,8) | 14.7 (13.5,16.3) | 0 (0,0) | 0.9 (0.4,1.5) | -95.1 (-97.7,-91.2) |
| Republic of Lithuania | 8 (7,9) | 9.3 (8.6,10) | 4 (2,7) | 0 (0,0.1) | -97.3 (-99.1,-94) |
| Republic of Singapore | 18 (17,19) | 2.1 (2,2.3) | 0 (0,1) | 0.1 (0,0.1) | -94.6 (-97.3,-90.6) |
| United Mexican States | 1623 (1475,1818) | 1.9 (1.7,2) | 86 (43,147) | 0.1 (0,0.1) | -95.3 (-97.7,-91.5) |
| Republic of Ecuador | 161 (144,178) | 2.4 (2.2,2.6) | 21 (4,57) | 0.1 (0,0.3) | -60.4 (-90.8,-6.2) |
| Principality of Andorra | 0 (0,0) | 3.1 (2.8,3.4) | 0 (0,0) | 0.3 (0.1,0.6) | -96.8 (-98.4,-94.6) |
| Republic of Austria | 15 (13,16) | 4.9 (4.6,5.2) | 1 (0,2) | 0.6 (0.2,1.2) | -99.6 (-99.9,-99.1) |
| Kingdom of Denmark | 19 (17,21) | 3.6 (3,4.1) | 1 (0,2) | 0.1 (0,0.1) | -98.9 (-99.6,-97.6) |
| Republic of Finland | 26 (23,29) | 2.1 (1.6,2.8) | 1 (0,1) | 0 (0,0.1) | -98.4 (-99.7,-95) |
| Republic of Iceland | 1 (1,1) | 2.7 (2.5,2.9) | 1 (1,2) | 0.1 (0,0.1) | -98.9 (-99.8,-97) |
| Kingdom of Belgium | 38 (34,41) | 4.1 (3.7,4.5) | 8 (3,16) | 0.1 (0,0.1) | -87.5 (-94,-80.1) |
| Republic of Italy | 101 (91,110) | 4.3 (3.8,4.7) | 9 (4,15) | 1.7 (0.8,2.5) | -73.2 (-84.3,-57.7) |
| Republic of Cyprus | 2 (1,2) | 3 (2.7,3.2) | 0 (0,0) | 0.3 (0.1,0.6) | -97 (-99.7,-89.6) |
| Hellenic Republic | 23 (21,24) | 2 (1.9,2.2) | 0 (0,1) | 0 (0,0) | -94.9 (-97.8,-90.3) |
| Grand Duchy of Luxembourg | 1 (1,1) | 4 (3.7,4.2) | 0 (0,0) | 0.2 (0,0.7) | -96.5 (-98.2,-93.9) |
| Republic of Malta | 1 (1,1) | 2.2 (2,2.3) | 0 (0,0) | 0.1 (0,0.1) | -84 (-93.1,-69.1) |
| State of Israel | 9 (9,10) | 2.7 (2.4,2.9) | 1 (0,2) | 0.1 (0,0.4) | -94.6 (-97.3,-90.6) |
| Portuguese Republic | 36 (33,38) | 1.5 (1.4,1.6) | 6 (1,19) | 0 (0,0.1) | -99.7 (-100,-98.9) |
| Republic of Angola | 737 (558,952) | 3.3 (3,3.6) | 369 (166,609) | 0.1 (0,0.1) | -89.8 (-96.5,-78.7) |
| Kingdom of Norway | 30 (26,33) | 9.2 (8.6,9.7) | 1 (0,3) | 0.1 (0,0.2) | -74 (-84.4,-59.7) |
| Republic of Chile | 108 (102,114) | 4.5 (4.1,4.8) | 2 (1,5) | 0.2 (0.1,0.3) | -74.6 (-92,-56) |
| Republic of Honduras | 106 (87,125) | 13.3 (11.1,15.7) | 21 (6,48) | 2.2 (0.6,5.1) | -98.3 (-99.5,-96) |
| Federative Republic of Brazil | 2020 (1804,2245) | 13.9 (12.5,15.4) | 260 (76,584) | 1.3 (0.4,2.8) | -89.3 (-97.4,-74.5) |
| Kingdom of the Netherlands | 46 (41,51) | 3 (2.8,3.3) | 4 (1,12) | 0.1 (0.1,0.2) | -99 (-99.8,-97.4) |
| Kingdom of Bahrain | 3 (2,3) | 4.3 (3.8,4.7) | 1 (0,1) | 0.1 (0,0.2) | -77.4 (-92.2,-55.2) |
| Kingdom of Sweden | 46 (40,51) | 8.3 (7.1,9.5) | 7 (2,16) | 0 (0,0.2) | -99.5 (-99.8,-97.6) |
| Swiss Confederation | 31 (27,34) | 4.7 (4.4,5) | 1 (1,2) | 0.2 (0.1,0.4) | -80.5 (-91.7,-59.4) |
| United Kingdom of Great Britain and Northern Ireland | 359 (322,392) | 13 (11.8,14.2) | 21 (11,36) | 1.4 (0.3,3.7) | -99.6 (-99.9,-98.2) |
| Kingdom of Bhutan | 25 (17,34) | 30.6 (26.6,35.5) | 3 (1,5) | 7.8 (2.5,13.2) | -98.3 (-99.2,-96.9) |
| Republic of India | 28106 (22399,33705) | 4.9 (4.2,5.7) | 8294 (3079,15045) | 0 (0,0.2) | -90.7 (-96,-81.9) |
| Eastern Republic of Uruguay | 14 (13,15) | 6.4 (5.5,7.4) | 1 (0,2) | 0 (0,0.1) | -95 (-99.4,-83.2) |
| Commonwealth of the Bahamas | 1 (1,2) | 36.9 (29.7,45.6) | 0 (0,0) | 0.1 (0,0.5) | -98.8 (-99.5,-97.3) |
| Kingdom of Spain | 98 (88,107) | 5.7 (5.1,6.3) | 12 (3,33) | 0 (0,0.1) | -71.2 (-85.6,-48.9) |
| Republic of Guyana | 8 (7,10) | 24.7 (19.9,29.5) | 0 (0,0) | 7.9 (2.9,14.2) | -99.6 (-99.9,-99.1) |
| Republic of Haiti | 407 (324,510) | 38.8 (28.6,48.9) | 1 (0,7) | 0.4 (0.1,0.9) | -62.5 (-76.9,-41.8) |
| Plurinational State of Bolivia | 384 (278,490) | 5.9 (5.1,6.9) | 4 (1,10) | 0.8 (0.3,1.5) | -89.5 (-94.4,-82.2) |
| Republic of Costa Rica | 17 (16,19) | 8.2 (6.7,9.6) | 0 (0,1) | 0 (0,0.2) | -98.5 (-99.4,-96.9) |
| Commonwealth of Dominica | 0 (0,0) | 9.2 (8.1,10.3) | 0 (0,0) | 0.5 (0.1,1.2) | -95.5 (-97.9,-92.1) |
| Republic of Suriname | 3 (3,4) | 24.8 (22.9,27.2) | 0 (0,0) | 3.9 (1.5,8) | -84.1 (-93.8,-68.7) |
| Republic of Trinidad and Tobago | 6 (5,6) | 11 (9.3,12.8) | 0 (0,0) | 0.1 (0,0.3) | -97.1 (-98.6,-94.6) |
| Republic of Colombia | 367 (319,418) | 15.6 (13.4,18.2) | 19 (4,49) | 0.9 (0.3,1.7) | -97 (-98.5,-95) |
| Arab Republic of Egypt | 3642 (3008,4496) | 6.1 (5.3,6.9) | 304 (99,648) | 2.4 (0.5,5.7) | -81.5 (-92.6,-63.4) |
| Islamic Republic of Iran | 839 (693,1107) | 36.6 (28.4,46.6) | 92 (48,133) | 8.8 (4.2,13.9) | -78.1 (-89.6,-62.6) |
| Republic of El Salvador | 82 (68,96) | 5.1 (4.7,5.5) | 1 (0,2) | 0.1 (0,0.2) | -98 (-99,-96.7) |
| Republic of Guatemala | 311 (280,346) | 42.4 (35.2,52) | 55 (21,111) | 2.9 (1,6.2) | -81.8 (-92.7,-61.4) |
| Republic of Nicaragua | 99 (85,116) | 11.6 (9.7,15) | 5 (2,11) | 1.4 (0.7,2.1) | -63.6 (-82.6,-34.5) |
| Republic of Panama | 16 (13,18) | 8 (6.5,9.9) | 9 (2,21) | 0.5 (0.1,1.2) | -98.9 (-99.8,-96.9) |
| Bolivarian Republic of Venezuela | 159 (149,171) | 7.1 (6.6,7.5) | 17 (9,29) | 0.7 (0.4,1.2) | -89.7 (-95.6,-79) |
| Republic of Paraguay | 45 (36,57) | 10.6 (8.1,14.1) | 3 (1,8) | 1.1 (0.5,2.1) | -89.9 (-95.8,-79.6) |
| People's Democratic Republic of Algeria | 351 (259,482) | 12 (9.5,15.1) | 43 (20,79) | 1.2 (0.5,2.3) | -65.6 (-80.5,-42.5) |
| Republic of Yemen | 721 (546,1017) | 8.9 (7.4,10.4) | 97 (43,180) | 1.1 (0.3,2.6) | -89.3 (-95.3,-78.9) |
| People's Republic of Bangladesh | 6581 (5522,7768) | 36.9 (28.6,45.5) | 743 (325,1378) | 6.7 (4.3,10.1) | -95.7 (-99.3,-87) |
| Federal Democratic Republic of Nepal | 1663 (1352,2009) | 20.1 (15.4,26.8) | 153 (57,309) | 1 (0.5,1.9) | -81.3 (-92.7,-63.3) |
| Islamic Republic of Pakistan | 4728 (3744,5841) | 22.5 (17.3,28.6) | 2368 (1308,3811) | 1.5 (0.6,2.8) | -96.9 (-98.6,-94.5) |
| State of Kuwait | 9 (8,10) | 27.9 (21.2,35.1) | 5 (2,9) | 11.3 (7.1,17.7) | -99.5 (-99.9,-98.6) |
| State of Libya | 35 (27,47) | 6.4 (5,8.2) | 5 (2,11) | 1.2 (0.5,2.5) | -68.6 (-80.9,-49.8) |
| Lebanese Republic | 42 (30,57) | 9.8 (7.5,14) | 9 (4,17) | 1.4 (0.5,2.8) | -76.1 (-88.4,-61.2) |
| Republic of Tunisia | 151 (103,239) | 4.4 (3.6,5.4) | 10 (4,18) | 0.1 (0,0.3) | -96.8 (-98.4,-94.7) |
| Republic of Turkey | 1370 (1042,1840) | 47.4 (38.6,57.1) | 66 (30,119) | 5.2 (1.9,10.4) | -99.7 (-99.9,-98.8) |
| Islamic Republic of Afghanistan | 785 (600,1045) | 18.6 (13.6,24.5) | 162 (71,321) | 6.7 (3.2,10.4) | -74 (-83.9,-59.5) |
| Kingdom of Morocco | 816 (624,1039) | 24.1 (20.3,29.9) | 46 (19,88) | 7.8 (4.8,11.8) | -88.1 (-95.3,-75.8) |
| State of Qatar | 2 (1,2) | 25.3 (19.4,35.1) | 0 (0,0) | 2.3 (1.1,4.3) | -99.4 (-99.8,-97.1) |
| Sultanate of Oman | 28 (20,42) | 24.6 (19.7,30.2) | 4 (2,8) | 8.3 (4.6,13.3) | -71.7 (-82.3,-57.2) |
| Republic of Iraq | 376 (297,478) | 5.9 (5.3,6.5) | 47 (20,88) | 2 (0.9,3.8) | -91.2 (-96,-82.9) |
| Hashemite Kingdom of Jordan | 49 (41,59) | 41.2 (31.9,54.4) | 10 (3,24) | 3.2 (1.4,6.2) | -94.9 (-97.6,-90.7) |
| Kingdom of Saudi Arabia | 190 (146,255) | 30.8 (22.2,40.3) | 20 (8,37) | 6.7 (3.1,11.2) | -99.7 (-99.9,-98.5) |
| Republic of Madagascar | 810 (697,940) | 11.1 (8.2,14.7) | 293 (102,579) | 1.8 (0.9,3.3) | -99.6 (-99.8,-99) |
| Republic of Kenya | 987 (815,1267) | 9.5 (7.6,12.2) | 355 (218,532) | 1 (0.4,1.8) | -93.4 (-97.2,-87) |
| Federal Democratic Republic of Ethiopia | 3526 (2657,4441) | 18.9 (13.8,24.7) | 884 (573,1329) | 7.1 (3.2,11.9) | -95.4 (-98.4,-90.5) |
| State of Eritrea | 162 (123,207) | 36.5 (31.8,42) | 83 (51,127) | 8.2 (2.9,16.4) | -93.8 (-97.3,-88.1) |
| Republic of the Congo | 66 (46,90) | 19 (15.9,23) | 30 (14,48) | 5.6 (2.1,10.5) | -78 (-91.6,-55.6) |
| Gabonese Republic | 27 (19,37) | 15 (10.4,23.4) | 11 (5,19) | 1 (0.4,1.9) | -61.8 (-78.9,-35) |
| Republic of Djibouti | 13 (9,17) | 34.8 (26.8,44.8) | 7 (4,11) | 9.1 (5.8,13) | -96.4 (-98.6,-92.7) |
| Republic of Equatorial Guinea | 26 (18,34) | 34.3 (29,40.3) | 8 (3,14) | 5.5 (2.4,10.2) | -70.9 (-81.5,-54.9) |
| Republic of Mozambique | 651 (480,956) | 29.4 (21.7,37) | 310 (191,480) | 7.9 (4.8,12.8) | -70.7 (-88.7,-43.6) |
| Republic of Botswana | 27 (21,33) | 20.6 (15.5,26.4) | 6 (2,12) | 6.5 (4,9.9) | -96.4 (-98.3,-93.7) |
| Kingdom of Lesotho | 42 (35,52) | 37.8 (28.4,48.2) | 10 (4,20) | 8.2 (5.1,12.5) | -62.4 (-81.8,-39.3) |
| Republic of Namibia | 28 (22,35) | 10 (9,11.2) | 6 (2,12) | 0.5 (0.3,0.9) | -79.2 (-87.6,-67.6) |
| Republic of South Africa | 1026 (882,1226) | 25.5 (17.2,34.2) | 126 (51,253) | 4.7 (2.1,8.7) | -87.3 (-96.4,-70) |
| Republic of the Gambia | 53 (42,67) | 36.9 (27.5,47.6) | 22 (14,32) | 10.4 (6.2,15.7) | -83.2 (-95.1,-59.6) |
| Republic of Burundi | 307 (223,400) | 7.4 (7,7.9) | 137 (77,229) | 0.3 (0.2,0.5) | -86.2 (-94.2,-72.6) |
| Republic of Rwanda | 494 (365,638) | 26.4 (19.8,37.9) | 122 (73,186) | 7.6 (4.7,11.4) | -79.9 (-88.6,-66.9) |
| Republic of Uganda | 633 (444,906) | 35.7 (25.6,47.4) | 347 (128,742) | 9.3 (5.8,14.7) | -94.8 (-98.8,-86.3) |
| Republic of Seychelles | 1 (1,1) | 14.6 (11.7,18.1) | 0 (0,0) | 2.6 (1,5.2) | -61.2 (-80.2,-41) |
| Republic of Zambia | 543 (428,696) | 22.4 (19.5,26.6) | 274 (153,418) | 2.7 (1.1,5.4) | -98.2 (-99.1,-96.7) |
| Republic of Malawi | 723 (526,960) | 16.1 (12.9,19.7) | 238 (137,365) | 3 (1.1,5.8) | -97 (-98.5,-94.9) |
| Union of the Comoros | 29 (23,38) | 21.9 (19.1,25.4) | 7 (4,10) | 4.8 (1.9,9.5) | -68.1 (-88.3,-42.5) |
| Republic of Mauritius | 7 (6,7) | 18.7 (15.3,22.5) | 0 (0,1) | 3.5 (1.4,6.9) | -99.2 (-99.8,-96.1) |
| Federal Republic of Somalia | 557 (387,752) | 18.2 (13.4,25.6) | 337 (205,543) | 6.2 (2.3,12.6) | -59.5 (-77.2,-36.4) |
| Kingdom of Eswatini | 23 (19,29) | 35.1 (28,44.3) | 4 (2,8) | 11.6 (6.7,17.5) | -90.9 (-97.4,-79.2) |
| Republic of Zimbabwe | 333 (284,390) | 40.1 (32.3,48.8) | 92 (36,181) | 15.4 (9.8,22.9) | -98.8 (-99.7,-96.4) |
| Republic of Benin | 357 (281,441) | 36.8 (29.5,45.1) | 237 (136,365) | 11.3 (6.5,17.1) | -95.8 (-97.9,-92.9) |
| Republic of Chad | 515 (407,635) | 35.3 (27.8,46) | 529 (332,796) | 7.2 (3.6,11.7) | -80.2 (-87,-69.4) |
| Republic of Cte d'Ivoire | 808 (622,1072) | 29.3 (23,37.7) | 280 (143,464) | 10.7 (5,18.1) | -94 (-97.3,-89.4) |
| Republic of the Niger | 1150 (864,1505) | 11 (8.6,14.1) | 618 (359,944) | 4.2 (2.6,6.7) | -61.6 (-76.2,-43.4) |
| Republic of Guinea | 701 (535,891) | 30.2 (24.7,37.6) | 241 (137,367) | 8.1 (5.2,12.1) | -79.8 (-90.1,-63.8) |
| Republic of Ghana | 491 (381,627) | 66.3 (49.9,86) | 256 (139,416) | 13.1 (7.7,19.8) | -66.4 (-84.1,-34.7) |
| Republic of Cameroon | 569 (438,743) | 20.6 (16.4,25.9) | 450 (205,778) | 7.1 (4.1,11.3) | -82.4 (-93.1,-65.3) |
| Republic of Cabo Verde | 6 (4,8) | 54 (44,64.1) | 2 (1,3) | 16.1 (9.3,24.6) | -79.1 (-87.5,-66.9) |
| Republic of Guinea-Bissau | 62 (47,79) | 57.9 (44.3,73.9) | 17 (11,26) | 12.1 (7,18.2) | -94.2 (-97.1,-90) |
| Republic of Liberia | 218 (164,287) | 29.4 (24,34.6) | 41 (24,65) | 5.8 (3.5,9.3) | -89 (-95.9,-77.2) |
| Republic of Senegal | 397 (321,477) | 34.8 (27.1,43.5) | 157 (79,279) | 7.3 (4.6,10.8) | -81.8 (-89,-72.5) |
| Republic of Sierra Leone | 407 (300,540) | 46.1 (34.6,60.4) | 117 (67,195) | 6.6 (4,10.3) | -69.4 (-81.7,-53.2) |
| Togolese Republic | 194 (153,244) | 27.5 (22.5,33) | 136 (74,213) | 7.9 (4.1,13.8) | -99.1 (-99.7,-97.8) |
| Republic of Mali | 418 (329,532) | 49.8 (36.9,65.2) | 381 (228,569) | 10 (5.8,16.4) | -93.3 (-97.1,-86.1) |
| Islamic Republic of Mauritania | 84 (67,102) | 23.9 (18.9,30.2) | 36 (23,55) | 9 (5.4,13.4) | -52.6 (-72.6,-24.1) |
| Democratic Republic of Sao Tome and Principe | 6 (4,7) | 2.9 (2.2,3.5) | 1 (1,2) | 0.2 (0.1,0.3) | -66.1 (-87.1,-31.1) |
| Federal Republic of Nigeria | 9113 (7429,10811) | 30.1 (24.1,36.9) | 5774 (3300,8860) | 14.3 (8,21.8) | -85.9 (-95,-71.8) |
| Principality of Monaco | 0 (0,0) | 18.1 (14.6,21.8) | 0 (0,0) | 1.1 (0.5,2.1) | -79.7 (-89,-66.1) |
| Republic of San Marino | 0 (0,0) | 23.8 (19.5,28.5) | 0 (0,0) | 6.9 (4.4,10.3) | -71.3 (-82,-52.3) |
| Republic of South Sudan | 394 (304,520) | 12.6 (10.2,15.4) | 201 (117,357) | 2.5 (1.3,4.2) | -70.1 (-82.1,-54.6) |
| Republic of Niue | 0 (0,0) | 15.4 (11.7,19.6) | 0 (0,0) | 0.7 (0.3,1.2) | -93.4 (-97,-87.1) |
| Republic of Nauru | 0 (0,0) | 2 (1.6,2.4) | 0 (0,0) | 0 (0,0.1) | -85.6 (-90.9,-77.5) |
| Republic of Palau | 0 (0,0) | 37.6 (29.3,48.9) | 0 (0,0) | 13.4 (7.9,22.6) | -64.5 (-78.3,-41.9) |
| Republic of Sudan | 1130 (830,1544) | 30.7 (22.8,41.9) | 100 (44,188) | 2 (0.9,3.7) | -80.2 (-88.6,-66.4) |

**Table S3. Joinpoint regression results of global in terms of age-standardized DALYs and deaths rate**

|  | **Both** | | |  | **Male** | | |  | **Female** | | |
| --- | --- | --- | --- | --- | --- | --- | --- | --- | --- | --- | --- |
|  | **Segments** | **APC** | **AAPC** |  | **Segments** | **APC** | **AAPC** |  | **Segments** | **APC** | **AAPC** |
| Deaths | 1990-2008 | -2.27 (-2.38 to -2.16) * | -5.38 (-5.84 to  -4.92) * |  | 1990-2008 | -2.22 (-2.33 to -2.11) * | -5.25 (-5.69 to  -4.80) * |  | 1990-2008 | -2.30 (-2.41 to -2.19) * | -5.48 (-5.92 to -5.05) * |
|  | 2008-2019 | -0.67 (-0.96 to -0.39) * |  |  | 2008-2019 | -0.60 (-0.89 to -0.31) * |  |  | 2008-2019 | -0.74 (-1.01 to -0.47) * |  |
|  | 2019-2021 | -45.83 (-49.84 to -41.49) * |  |  | 2019-2021 | -45.10 (-49.01 to -40.89) * |  |  | 2019-2021 | -46.42 (-50.20 to -42.36) * |  |
| DALYs | 1990-2008 | -2.48 (-2.59 to -2.38) * | -5.40 (-5.82 to  -4.97) * |  | 1990-2008 | -2.50 (-2.61 to -2.40) * | -5.27 (-5.68 to  -4.85)* |  | 1990-2008 | -2.47 (-2.57 to -2.36) * | -5.52 (-5.93 to -5.10) * |
|  | 2008-2019 | -0.79 (-1.07 to -0.51) * |  |  | 2008-2019 | -0.74 (-1.03 to -0.46) * |  |  | 2008-2019 | -0.84 (-1.10 to -0.58) * |  |
|  | 2019-2021 | -44.53 (-48.37 to -40.41) * |  |  | 2019-2021 | -43.43 (-47.21 to -39.38) * |  |  | 2019-2021 | -45.59 (-49.23 to -41.70) * |  |

*Represents that APC is significantly different from 0 at α=0.05 level; *Represents that AAPC is significantly different from 0 at α=0.05 level. DALYs: disability-adjusted life years
